# Beneficial effects of vascular endothelial growth factor B gene transfer in the aged heart

**DOI:** 10.1093/cvr/cvaf046

**Published:** 2025-03-21

**Authors:** Nivethitha Manickam, Ibrahim Sultan, Josefine Panthel, Haris Kujundzic, Ariane Fischer, Katja Schmitz, Mariano Ruz Jurado, David Rodriguez Morales, David John, Simone-Franziska Glaser, Kathrin A Stilz, Pedro Felipe Malacarne, Ralf Peter Brandes, Thomas Braun, Carolin Lerchenmüller, Fynn Betge, Wesley T Abplanalp, Kari Alitalo, Stefanie Dimmeler, Julian U G Wagner

**Affiliations:** Centre for Molecular Medicine, Institute of Cardiovascular Regeneration, Goethe University Frankfurt, Theodor Stern Kai 7, Building 25B, Frankfurt 60590, Germany; Translational Cancer Medicine Program, University of Helsinki, Helsinki 00290, Finland; Wihuri Research Institute, Biomedicum Helsinki, Helsinki 00290, Finland; Centre for Molecular Medicine, Institute of Cardiovascular Regeneration, Goethe University Frankfurt, Theodor Stern Kai 7, Building 25B, Frankfurt 60590, Germany; Centre for Molecular Medicine, Institute of Cardiovascular Regeneration, Goethe University Frankfurt, Theodor Stern Kai 7, Building 25B, Frankfurt 60590, Germany; Centre for Molecular Medicine, Institute of Cardiovascular Regeneration, Goethe University Frankfurt, Theodor Stern Kai 7, Building 25B, Frankfurt 60590, Germany; Centre for Molecular Medicine, Institute of Cardiovascular Regeneration, Goethe University Frankfurt, Theodor Stern Kai 7, Building 25B, Frankfurt 60590, Germany; Centre for Molecular Medicine, Institute of Cardiovascular Regeneration, Goethe University Frankfurt, Theodor Stern Kai 7, Building 25B, Frankfurt 60590, Germany; German Center for Cardiovascular Research (DZHK), Partner Site Rhein-Main, Frankfurt 60590, Germany; Cardiopulmonary Institute (CPI), Frankfurt 60590, Germany; Centre for Molecular Medicine, Institute of Cardiovascular Regeneration, Goethe University Frankfurt, Theodor Stern Kai 7, Building 25B, Frankfurt 60590, Germany; German Center for Cardiovascular Research (DZHK), Partner Site Rhein-Main, Frankfurt 60590, Germany; Cardiopulmonary Institute (CPI), Frankfurt 60590, Germany; Centre for Molecular Medicine, Institute of Cardiovascular Regeneration, Goethe University Frankfurt, Theodor Stern Kai 7, Building 25B, Frankfurt 60590, Germany; German Center for Cardiovascular Research (DZHK), Partner Site Rhein-Main, Frankfurt 60590, Germany; Cardiopulmonary Institute (CPI), Frankfurt 60590, Germany; Centre for Molecular Medicine, Institute of Cardiovascular Regeneration, Goethe University Frankfurt, Theodor Stern Kai 7, Building 25B, Frankfurt 60590, Germany; German Center for Cardiovascular Research (DZHK), Partner Site Rhein-Main, Frankfurt 60590, Germany; Cardiopulmonary Institute (CPI), Frankfurt 60590, Germany; Centre for Molecular Medicine, Institute of Cardiovascular Regeneration, Goethe University Frankfurt, Theodor Stern Kai 7, Building 25B, Frankfurt 60590, Germany; Centre for Molecular Medicine, Institute for Cardiovascular Physiology, Goethe University Frankfurt, Frankfurt 60590, Germany; German Center for Cardiovascular Research (DZHK), Partner Site Rhein-Main, Frankfurt 60590, Germany; Cardiopulmonary Institute (CPI), Frankfurt 60590, Germany; Centre for Molecular Medicine, Institute for Cardiovascular Physiology, Goethe University Frankfurt, Frankfurt 60590, Germany; Cardiopulmonary Institute (CPI), Frankfurt 60590, Germany; Centre for Molecular Medicine, Institute for Cardiovascular Physiology, Goethe University Frankfurt, Frankfurt 60590, Germany; Max Planck Institute for Heart and Lung Research, Bad Nauheim 61231, Germany; Chair of Gender Medicine, University of Zurich, Zurich 8006, Switzerland; Department of Cardiology, University Hospital Zurich, Zurich 8091, Switzerland; Department of Cardiology, University Hospital Heidelberg, Heidelberg 69120, Germany; German Center for Cardiovascular Research (DZHK), Partner Site Heidelberg/Mannheim, Heidelberg 69120, Germany; Department of Cardiology, University Hospital Heidelberg, Heidelberg 69120, Germany; German Center for Cardiovascular Research (DZHK), Partner Site Heidelberg/Mannheim, Heidelberg 69120, Germany; Centre for Molecular Medicine, Institute of Cardiovascular Regeneration, Goethe University Frankfurt, Theodor Stern Kai 7, Building 25B, Frankfurt 60590, Germany; German Center for Cardiovascular Research (DZHK), Partner Site Rhein-Main, Frankfurt 60590, Germany; Cardiopulmonary Institute (CPI), Frankfurt 60590, Germany; Translational Cancer Medicine Program, University of Helsinki, Helsinki 00290, Finland; Wihuri Research Institute, Biomedicum Helsinki, Helsinki 00290, Finland; Centre for Molecular Medicine, Institute of Cardiovascular Regeneration, Goethe University Frankfurt, Theodor Stern Kai 7, Building 25B, Frankfurt 60590, Germany; German Center for Cardiovascular Research (DZHK), Partner Site Rhein-Main, Frankfurt 60590, Germany; Cardiopulmonary Institute (CPI), Frankfurt 60590, Germany; Centre for Molecular Medicine, Institute of Cardiovascular Regeneration, Goethe University Frankfurt, Theodor Stern Kai 7, Building 25B, Frankfurt 60590, Germany; German Center for Cardiovascular Research (DZHK), Partner Site Rhein-Main, Frankfurt 60590, Germany; Cardiopulmonary Institute (CPI), Frankfurt 60590, Germany

**Keywords:** Heart, Ageing, VEGFB

## Abstract

**Aims:**

Members of the VEGF family are crucial modulators of vascular and neural function. While VEGFA signalling has been shown to mitigate several aging-related cardiac phenotypes and prolong survival in aged mice, the role of VEGFB in cardiac aging remains underexplored. In this study, we identify a significant decline in *Vegfb* expression, particularly of its soluble isoform *Vegfb_186_*, in aged mouse and human hearts. To assess the therapeutic potential of VEGFB in aging-associated cardiac pathologies, we used AAV9-mediated gene transfer to overexpress *Vegfb_186_* in 18-month-old male C57Bl/6J mice.

**Methods and results:**

VEGFB is known to exhibit vascular and neuroprotective effects that we assessed in the ageing heart. In the aged heart, doses of *Vegfb_186_* overexpression that had only a modest effect on the vascular endothelium prevented age-induced diastolic dysfunction and fibrosis. Vegfb186 treatment additionally restored sympathetic and sensory nerve fibre density and increased heart rate variability. Although *Vegfb_186_* overexpression induced cardiac hypertrophy, our findings indicated that this hypertrophy was compensatory rather than pathological as *Vegfb_186_* overexpression corrected the elevated cardiomyocyte length-to-width ratio observed in aged hearts, a metric typically indicative of pathological remodelling. Cardiac single-nucleus RNA sequencing of the hearts and *in vitro* analysis of the cardiomyocytes indicated up-regulation of the STAT3 signal transduction pathway as a potential contributor of VEGFB-induced cardiac hypertrophy.

**Conclusion:**

Our findings demonstrate that *Vegfb_186_* overexpression partially reverses age-related cardiac pathologies such as diastolic dysfunction and fibrosis. This work highlights VEGFB as a potential therapeutic target for combating cardiac aging and its associated dysfunctions.

## Graphical Abstract

**Graphical Abstract:**
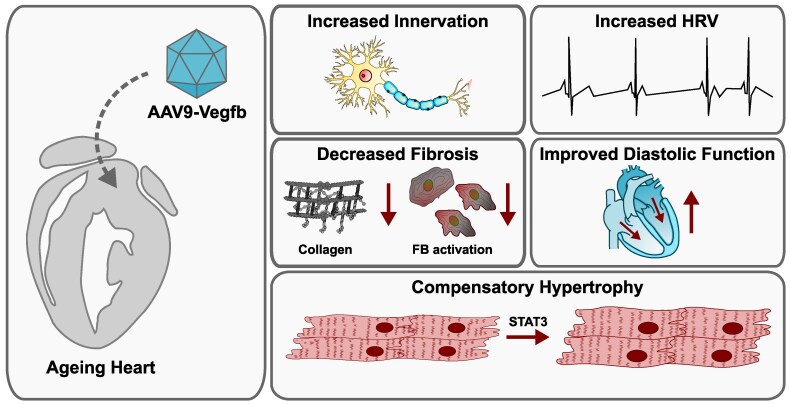

**Time of primary review: 57 days**

## 1. Introduction

Ageing represents a major risk factor for cardiovascular diseases, which still form the leading global cause of mortality.^1^ As populations with rising life expectancies continue to age, cardiovascular diseases have emerged as an escalating global health challenge. Despite remarkable strides in cardiovascular research and therapeutic interventions, the intricate molecular, and cellular processes governing cardiac ageing and its associated pathologies remain understudied.

In the context of ageing, the vasculature has attracted particular attention due to its pivotal role in maintaining tissue homeostasis and orchestrating tissue regeneration across various organ systems. The heart critically depends on a functional microvasculature to ensure a continuous supply of oxygen for the high energy demands of contracting cardiomyocytes. Furthermore, vascular niches have been recognized as a sources of ‘angiocrine’ factors, which have risen to prominence as pivotal paracrine regulators governing tissue homeostasis and repair.^2–8^ An impaired vascular function together with cardiomyocyte death or hypertrophy, plus fibrosis, represent the hallmarks of ageing-related changes in the heart.^9,10^ Hence, the preservation of a functional vasculature is essential for prevention or mitigation of cardiac ageing.

Among the many regulatory molecules governing vascular health, the family of vascular endothelial growth factors (VEGFs) consists of well-established orchestrators of endothelial cell functions. Various members of this family, such as VEGFA and VEGFC, are renowned for their roles in promoting angiogenesis and vascularization by blood and lymphatic vessels, respectively, via activation of their corresponding VEGF receptors (VEGFRs).^11,12^ Recently, low-dose VEGFA was used to extend the lifespan in mice and to rejuvenate numerous organs.^13^ VEGFB which exhibits lower angiogenic potency than VEGFA, can function as a coronary endothelial growth factor, promoting endocardial-derived collateralization and cardiac regeneration without significant vascular leakage, or inflammation.^14,15^ Furthermore, VEGFB gene therapy has been found to support endothelial cell survival^16^ and induce metabolic reprogramming of the heart, characterized by reduced lipoprotein lipase activity and improved insulin action.^17,18^ Although the regulatory mechanisms and functional implications of VEGFB in the context of ageing remain enigmatic, recent investigations have unveiled a striking down-regulation of VEGFB expression in diseased myocardium in both humans and mice.^17,19,20^ This observation raises the possibility that the reduction of VEGFB may contribute to the pathogenesis in cardiac diseases. The obvious question therefore is if *Vegfb* overexpression has the potential to alleviate ageing-associated decline of cardiac tissue and function, especially as VEGFB can induce cardiac hypertrophy and neuronal growth that could increase cardiac vulnerability.^21,22^

In light of these considerations, we have here analysed if we can rescue age-related cardiac pathologies by VEGFB gene therapy.

## 2. Methods

### 2.1 Laboratory animals

Aged male C57Bl/6J wildtype mice were purchased from Janvier (Le Genest SaintIsle, France) and from Charles River (Sulzfeld, Germany). Homozygosity of these inbred mice was controlled by Janvier and Charles River using exome sequencing. All animal experiments have been executed in accordance to the guidelines from Directive 2010/63/EU of the European Parliament on the protection of animals used for scientific purposes and were approved by the local authorities by the state of Hesse (Regierungspräsidium Darmstadt). Experiments were conducted as follows:

### 2.2 Adeno associated viral vector (AAV9) delivery

To overexpress VEGFB_186_ in aged mouse hearts, AAV9 vectors carrying the VEGFB_186_ gene under the control of the CAG promoter were used. AAV9 particles without payload served as a negative control. The viral particles were injected intraperitoneally, at a concentration of 2.2 × 10^11^ viral particles per animal to 18-month-old mice. In the heart, the AAV9 vectors were expressed mainly in cardiomyocytes, as previously demonstrated by Räsänen *et al*. (see Supplementary material online, *Figure S3*) and by Sultan *et al*. (see Supplementary material online, *Figure S12F*, *G*, *H*).^15,23^

Before AAV9-treatment (baseline) and 4 and 8 weeks after the treatment, echocardiography was used to monitor the systolic and diastolic function of each mouse.

### 2.3 Telemetric ECG recordings

To record long-term ECG traces remotely in awake mice, ETA-F10 transmitters (270–0160-002, DSI) were implanted subcutaneously as previously described.^24^ Sympathetic activity was analysed over 6 weeks using the software Ponemah 6 (DSI) by calculating the time and frequency domain parameters to assess heart rate variability. Heart rate variability was determined as time domain (SDNN) and frequency domain (LF/HF) measurements between 11.00 am and 1.30 pm (day) and between 12.00 am and 2.30 am.

### 2.4 Echocardiography

To assess heart function via echocardiography, mice were anaesthetized with 2–2.5% of isoflurane and monitored using the Vevo 3100 echocardiography system with the Vevo LAB software (Fujifilm VisualSonics), at a time point of 4 and 8 weeks after the administration of the AAVs.

### 2.5 Euthanasia

Mice were anaesthetized with 2–2.5% of isoflurane and euthanized via cervical dislocation.

### 2.6 Cell isolation from murine heart

Cardiac endothelial cells were isolated from young (3 months) and old (20 to 24 months) male mice as previously described.^24^ To isolate fibroblasts and cardiomyocytes, heart homogenates were centrifuged at 80 *g* for 5 min. Each pellet was resuspended in DMEM supplemented with 5% glucose (11965092, Gibco) and 10% FBS (A5209502, Gibco) and plated in one well of a 12-well plate and incubated for 2 h at 37°C and 5% CO_2_ at humidified atmosphere. Cardiomyocytes do not adhere to plastic plates and were therefore collected from the supernatant, whereas fibroblasts adherent to plastic and were collected from the plates.

### 2.7 Isolation of murine sympathetic neurons

The right and the left stellate ganglia were isolated as previously described^25^ and washed in ice cold PBS. They were then washed in ice cold calcium-free tyrode solution twice and digested with Liberase (Roche, 5401119001) and Elastase (Serva, 20930.01) in the same solution for 1 h at 37°C. The stellate ganglia suspension was again washed with calcium-containing tyrode solution twice and followed by Neurobasal medium (21103049, Gibco) containing GlutaMAX-I (35050, Gibco) and B-27 supplement (17504, Gibco) before plating. The neurons were maintained in culture for 72 h.

### 2.8 Aortic ring assay

Mice were euthanized and hearts were flushed with ice cold HBSS. The aorta was dissected cross-sectionally into at least eight rings. Each ring was embedded in 50 µL rat tail collagen type 1 (354236, Corning) containing 1 × medium M119 (M0650, Sigma-Aldrich), 8 mM NaHCO_3_ (HN01.2, Carl Roth GmbH), and 10 mM NaOH (6771.1 Carl Roth GmbH) in a 96 well plate. After adding 150 µL DMEM/F-12 (10565018, Gibco) supplemented with 2.5% FBS (4133, Invitrogen) per well, aortic rings were cultured in presence or absence of VEGFA (SRP4363-10UG, Sigma-Aldrich). After 7 days, the aortic rings were fixed with 4% PFA (28908, Thermo Scientific) for 30 min, permeabilized with 0.25% Triton-X containing PBS for 15 min. After blocking with Dako protein blocking buffer (X0909, DAKO), the aortic rings were incubated overnight in PBLEC buffer (50 µL of 1 M MgCl_2_, 50 µL of 1 M CaCl_2_, 5 µL of 1 M MnCl_2_, 5 mL of 10% Triton-X-100, in 50 mL PBS) containing biotinylated Isolectin B4 (VEC-B-1205, Biozol) at a concentration of 1:100. Rings were washed three times with PBS and stained with streptavidin-conjugated Alexa 488 (S32354, Invitrogen) for 3 h at room temperature. After washing with PBS three times (15 min each), the rings were imaged using Nikon Eclipse T i2-E.

### 2.9 ELISA

VEGFB protein levels were determined using the VEGFB mouse ELISA kit EMVEGFB (ThermoFisher) according to the manufacturer’s instruction.

### 2.10 Western blot

Protein expression in mouse hearts was analysed by immunoblotting. In brief, hearts were homogenized in 1 mL RIPA buffer (89900, Thermo Scientific) containing a ¼′ ceramic spheres two times for 20 s each at 4 m/s. After centrifugation (20 000 *g*, 4°C for 20 min), supernatants were collected. For immunoblotting, 30 µg protein lysate was combined with Laemmli buffer, treated for 5 min at 95°C and separated by SDS-PAGE. Proteins in gel were then transferred to a nitrocellulose membrane that was blocked using TBST (1061.1, Carl Roth GmbH & Co. KG) containing 5% skimmed milk (sc-281695, Santa Cruz). After blocking, the membranes were incubated in primary antibodies overnight at 4°C. The membranes were washed three times in TBST for 5 min each and incubated in blocking solution containing the Rb anti-IgG antibody conjugated to horseradish peroxidase (1:5000, NA934 V, Cytiva) for 1 h at room temperature. After washing three times for 5 min each with TBST, signal was visualized using the chemiluminescence HRP substrate ECL (WBKLS0500, Merck-Milipore).

### 2.11 Immunofluorescence staining of paraffin sections

Paraffin sections were deparaffinized at 60°C for 30 min and rehydrated using RotiHistol (two times for 10 min) and a descending ethanol series (100%, 95%, 80, 70 and 50% ethanol; 5 min each). Sections were then washed with water for 5 min and incubated in boiling 0.01 M citrate buffer (pH 6.0) for 90 to 120 s. The slides were blocked with PBS containing 0.1% Triton-X100, 3% BSA (A7030-10G, Merck), and 5% donkey serum (ab7475, Abcam) and incubated with primary antibodies in the blocking solution overnight at 4°C. Afterwards, sections were washed three times in PBS and once in PBS containing 0.1% triton (5 min each) and incubated with the corresponding secondary antibodies and DAPI (6335.1, Carl Roth GmbH & Co.KG), diluted in PBS containing 5% BSA, for one hour at room temperature. The sections were washed with PBS two times for 5 min each and mounted with Fluoromount-G^TM^ (00-4958-02, Invitrogen). Images were taken using the Leica DMi8 Stellaris confocal microscope and the LASX software.

### 2.12 Immunofluorescence of cryopreserved sections

The hearts flushed with cold HBSS were fixed overnight at 4°C in 4% PFA in PBS (28908, ThermoFisher Scientific). The fixed hearts were washed with PBS three times, 10 min each wash. The hearts were then subjected to three consecutive overnight incubations at 4°C with increasing concentrations of sucrose (10%, 20%, and 30%; S0389, Sigma-Aldrich) in PBS and the tissues were embedded in 15% sucrose with 8% gelatin (G1890, Sigma-Aldrich), and 1% polyvinylpyrrolidone (P5288, Sigma-Aldrich) in PBS. The embedded tissues were allowed to solidify and stored at −80°C. They were then sectioned using the Leica cryostat CM3050 S into 50 µm sections, mounted on adhesive glass slides (10149870, ThermoFischer Scientific), dried overnight at room temperature and stored at −20°C.

For immunofluorescence staining, sections were allowed to thaw at room temperature for 5 min, and washed with PBS twice (5 min each), three times with PBS containing 0.3% Triton X-100 (10 min each) and blocked with 0.1% Triton X-100, 3% BSA (A7030-10G, Merck), and 5% donkey serum (ab7475, Abcam) in PBS for 1 h at room temperature. After blocking, the sections were incubated with primary antibodies in blocking solution, overnight at 4°C. The slides were washed with PBS three times (5 min each), and, after one wash with 0.1% Triton X-100 in PBS, incubated with corresponding secondary antibody in PBS containing 5% BSA (A7030-10G, Merck) for 1 h at room temperature. The slides were then washed two times with PBS for 5 min and mounted with Fluoromount-G^TM^ (00-4958-02, Invitrogen). Images were captured using the Leica DMi8 confocal Stellaris microscope and the LASX software.

### 2.13 Immunofluorescence staining of cultured cells

Cells were fixed with 4% PFA for 10 min at room temperature, washed with PBS containing 0.1% Triton-X for 10 min, and blocked using 2% donkey serum (ab7475, Abcam), 1% BSA (A7030-10G, Merck), and 0.1% Triton-X in PBS for 1 h, then incubated with the primary antibodies overnight at 4°C. The cells were washed with PBS, three times for 5 min and incubated with the corresponding secondary antibody for 1 h at room temperature, followed by PBS washes for three times, 5 min each, mounted with Fluoromount-G^TM^ (00-4958-02, Invitrogen), and imaged with Leica DMi8 confocal Stellaris microscope and the LASX software.

### 2.14 Antibodies used for immunofluorescence

The following primary antibodies were used: Rabbit anti-Tuj1 (1:100, Abcam, AB18207), Rabbit anti-TH (1:100, Millipore, AB152), Goat anti-CGRP (1:100, Abcam, ab36001), Rabbit anti-VEGFB (1:1000 for WB, PA5-116113, Invitrogen), Rabbit anti-ACHE (1:1000 for WB, ab97299, Abcam), Rat anti-a-tubulin (1:5000 for WB, ab6160, Abcam), Rabbit anti-GAPDH (1:1000 for WB, 2118, CST), Rabbit anti-STAT3 (1:1000 for WB, 12640, CST), Rabbit anti-phosphoTyr705-STAT3 (1:1000 for WB, 9145, CST), Chicken anti-MAP2 (1:100, Abcam, ab5392), Rabbit anti-CD68 (CST, 97778), Rat anti-CD45 (1:100, Millipore, 05-1416), Rabbit anti-cleaved caspase 3 (1:100, CST, 9664), and Mouse anti-α-smooth muscle actin—Cy3™ (αSMA, 1:200, C6198-2ML, Sigma-Aldrich).

The secondary antibodies were: Alexa Fluor^TM^ 488 Phalloidin (1:100, Invitrogen, A12379), Streptavidin Alexa Fluor™ 488 conjugate (1:100, Invitrogen, S32354), Donkey anti-rabbit antibody Alexa Fluor 488 (1:200, Invitrogen, A-21206), Donkey anti-Rabbit Secondary Antibody Alexa Fluor 555 (1:200, Invitrogen, A-31572), and Donkey anti-Chicken Alexa Fluor 488 (1:200, Dianova, 703-545-155).

Otherwise GSL 1-Isolectin B4 (IB4) (1:25, Biozol, VEC-B-1205) and Wheat Germ Agglutinin (WGA), Alexa Fluor™ 555 Conjugate (1:100, W32464, Invitrogen) were used. WGA was diluted together with the secondary antibodies as sera would block WGA binding.

### 2.15 Analysis of immunofluorescence images

The images were analysed using software Volocity 7 by Quorum Technologies Inc. and the quantified signals were normalized to IB4 or DAPI area.

### 2.16 Acidic beta galactosidase staining

The staining for cryopreserved heart sections was carried out using the senescence β-galactosidase staining kit (9860, CST) according to manufacturer’s instruction. The β-galactosidase positive areas were quantified using ImageJ.

### 2.17 Picrosirius red staining

Heart sections were deparaffinized using wash with RotiHistol, twice for 10 min each wash and rehydrated using an ethanol gradient series (100%, 95%, 80%, 70%, and 50% ethanol, 5 min each). After 5 min in water, the sections were incubated with 0.1% picrosirius red solution (0.5 g sirius red in 500 mL of saturated aqueous picric acid) for 1 h, then washed twice with acidified water. The stained slides were dehydrated using 100% ethanol, cleared with xylene and mounted using pertex mounting medium (R0080, Histoline).

### 2.18 RNA isolation

To isolate total RNA from cardiac tissue, the hearts were combined with 700 µL of Qiazol and ¼′ ceramic spheres and homogenized three times for 20 s at 4 min/s. The miRNeasy Mini kit (217004, Qiagen) was used for further steps of RNA isolation. The RNA concentration was determined by measuring absorption at 260 and 280 nm in a NanoDrop ND 2000-spectrophotometer (PeqLab).

### 2.19 cDNA synthesis and quantitative PCR

The reverse transcription was carried out using reverse transcriptase M-MLV (28025013, ThermoFisher Scientific) and 500 ng of RNA and assessed using the SYBR™ Green PCR Master Mix (4385617, Applied Biosystems). The primers were designed and purchased from Merck:

Mouse *Vegfa* fw (GCAGCGACAAGGCAGACTAT)

Mouse *Vegfa* rev (AACCTCCTCAAACCGTTGGC)

Mouse *Vegfb_167_* fw (GGCAACACCAAGTCCGAATG)

Mouse *Vegfb_167_* rev (GAGGATCCTGGGGCTGTCT)

Mouse *Vegfb_186_* fw (TCTGTTCCGGGCTGGGA)

Mouse *Vegfb_186_* rev (AGTGGGATGGATGATGTCAGCT)

Mouse *Nfkb* fw (GCTTAGGAGGGAGAGCCCAC)

Mouse *Nfkb* rev (AGGTATGGGCCATCTGCTGT)

Mouse *Il6* fw (AGACAGCCACTCACCTCTTC)

Mouse *Il6* rev (TTTCACCAGGCAAGTCTCCT)

Mouse *Tnf* fw (CCCTCACACTCAGATCATCTTCT)

Mouse *Tnf* rev (GCTACGACGTGGGCTACAG)

Mouse *Il10* fw (GCTCTTACTGACTGGCATGAG)

Mouse *Il10* rev (CGCAGCTCTAGGAGCATGTG)

Mouse *Rpl0* fw (GCGTCCTGGCATTGTCTGT)

Mouse *Rpl0* rev (GAAGGCCTTGACCTTTTCAGTAAG)

### 2.20 TaqMan PCR

To detect miR222-3p expression in murine hearts, cDNA was reverse-transcribed using the TaqMan MicroRNA Reverse Transcription Kit (4366596, ThermoFisher) and the TaqMan primers against U6 (4427975; ID: 001973, ThermoFisher) and against miR222-3p (4440418; ID: CTFVMKG, TermoFisher). TaqMan PCR was performed using the TaqMan Advanced kit (4427975, ThermoFisher) according to the manufacturer’s protocol.

### 2.21 Single nuclei RNA sequencing and data analysis

Single nuclei RNA sequencing and data assessment were performed as previously described.^24^

### 2.22 Cell culture

Primary mouse cortical neurons (A15585, Gibco) were purchased and cultured, according to manufacturer’s instructions, in Neurobasal medium (21103049, Gibco) containing GlutaMAX-I (35050, Gibco) and B-27 supplement (17504, Gibco). Neurons were treated either with conditioned HUVEC medium without additional VEGFB (50:50, Neurobasal^TM^ medium: conditioned medium) or with 100 ng/mL recombinant VEGFB_186_ (Biotechne, 767-VE-010)

Primary human cardiac fibroblasts (HCF-c) were purchased from Promocell (12375) and cultured according to manufacturer`s instructions in the fibroblast basal medium 3 (C-23230, Promocell) containing the recommended supplements (c-39350, Promocell) in 18 well Ibidi slides (81816, 15 µ-Slide). The fibroblasts were treated with either TGF beta (302-B2-10, Biotechne), VEGFB (767-VE-010, Biotechne) or both for 48 h.

Neonatal rat cardiomyocytes were isolated and maintained as previously described.^19^ The cardiomyocytes were subjected to phenylephrine (200 µM), VEGFB (100 ng/mL; 767-VE-010, Biotechne) or both for 72-h, with supplementation of fresh media every 24 h. To block STAT3 signalling, cardiomyocyte cultures were supplemented with 10 µM STAT3 inhibitor XIII (C188-9 Calbiochem) or 100 nM rapamycin (553210, Merck) for 72 h.

### 2.23 Statistical analysis

Data are represented as mean and error bars indicate standard error of the mean (SEM). Shapiro Wilk test was carried out for normality distribution analysis. Unpaired or paired two sided *t*-test, or Mann–Whitney *U* test and an ordinary one-way ANOVA with a *post hoc* Tukey test or a Kruskal–Wallis test was used for comparison of two groups and more than two groups respectively.

To assess snRNA sequencing data, *P*-values were calculated by using the Wilcox-test.

## 3. Results

### 3.1 *Vegfb* expression is reduced in aged mouse hearts

**Figure 1:**
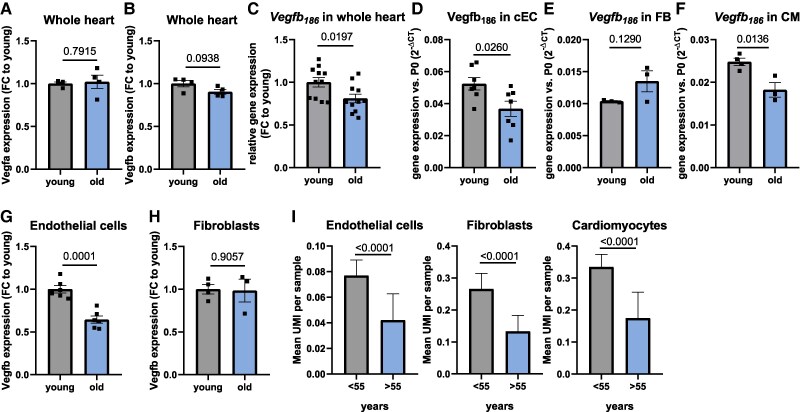
*Vegfb* gene expression is declined with age. (*A*, *B*) Published transcriptomic data of hearts derived from 2-month-old (young) vs. 18- to 20-month-old (old) mice^28^ (*n* = 4). Panel (*A*) shows *Vegfa* and panel (*B*) shows *Vegfb* expression. (*C*–*F*) *Vegfb* expression detected by RT-qPCR on whole heart tissues from 3-month-old (young) vs. 18- to 20-month-old (old) mice (*C*, *n* = 11), in isolated cardiac endothelial cells from 3-month-old (young) vs. 18− to 20-month-old (old) mice (*D*, *n* = 7), in isolated fibroblasts (FB, *E*, *n* = 3) and cardiomyocytes (CM) from 3-month-old (young) vs. 24-month-old (old) mice (*F*, *n* = 4 vs. *n* = 3). (*G*, *H*) *Vegfb* expression data derived from published bulk RNA sequencing data from isolated cardiac endothelial cells (*G*, *n* = 6)^24^ and cardiac fibroblasts (*H*, *n* = 4 vs. *n* = 3).^29^ (*I*) *VEGFB* expression in human cardiac endothelial cells, fibroblasts, and cardiomyocytes. Data are derived from published human cardiac snRNA sequencing data of LV samples from individuals being below or above 55 years old.^30^ Data are shown as mean and error bars indicate SEM. Data are Gaussian distributed and *P*-value was assessed by two-tailed, unpaired Student’s *t*-test (*A–H*). To calculate *P*-values in panel (*I*) (snRNA sequencing data), a Wilcox-test was used.

VEGFB is a secreted paracrine factor that is mainly produced by cardiomyocytes in the heart.^17,26,27^To study the cardiac expression of *Vegfb,* we analysed our published transcriptomics data from hearts of 2-month-old (young) and 20-month-old (old) mice.^28^ Unlike *Vegfa*, which was expressed at similar levels in young and old hearts (*Figure 1A*), *Vegfb* tended to be down-regulated with advanced age (*Figure 1B*). Quantitative RT-PCR on whole heart tissue and isolated cardiac endothelial cells from young (3 months) and old (20–24 months) mice confirmed the down-regulation of *Vegfb* during ageing (*Figure 1C and D*). While isolated fibroblasts showed no regulation of *Vegfb*, isolated cardiomyocytes from aged mice revealed a significant decline in *Vegfb* expression (*Figure 1E* and *F*). Bulk RNA sequencing data from cardiac endothelial cells^24^ and fibroblasts^29^ from previously published datasets confirmed a substantial reduction of *Vegfb* expression in endothelial cells isolated from old mouse hearts, but not in fibroblasts (*Figure 1G and H*). Single nuclei RNA sequencing (snRNA seq) data from human left ventricles^30^ further confirmed down-regulation of *VEGFB* mRNA expression in cardiac endothelial cells, fibroblasts and cardiomyocytes in individuals above 55 years (*Figure 1I*). In addition, we found a significant down-regulation of *VEGFB* and up-regulation of *VEGFA* in patients with pathological hypertrophy^19^ (see Supplementary material online, *Figure S1*).

Of the two distinct VEGFB isoforms, VEGFB_186_ encodes the soluble form, potentially facilitating paracrine signalling, while VEGFB_167_ encodes a variant that has high affinity to cell-surface and peri-cellular matrix.^26,31–33^ To distinguish between these isoforms, we conducted RT-qPCR analysis on whole hearts obtained from 3- vs. 20-month-old mice. Interestingly, we found that *Vegfb_186_* transcripts were decreased in the ageing mouse heart, while *Vegfb_167_* transcripts remained unchanged (see Supplementary material online, *Figure S2*). As previous studies have shown that VEGFB_186_ is more abundant than VEGFB_167_ upon similar mRNA expression,^23^ these results suggested that the paracrine signalling mediated by VEGFB is attenuated during cardiac aging.

### 3.2 Overexpression of *vegfb* using AAV9 vectors in aged mouse hearts

**Figure 2:**
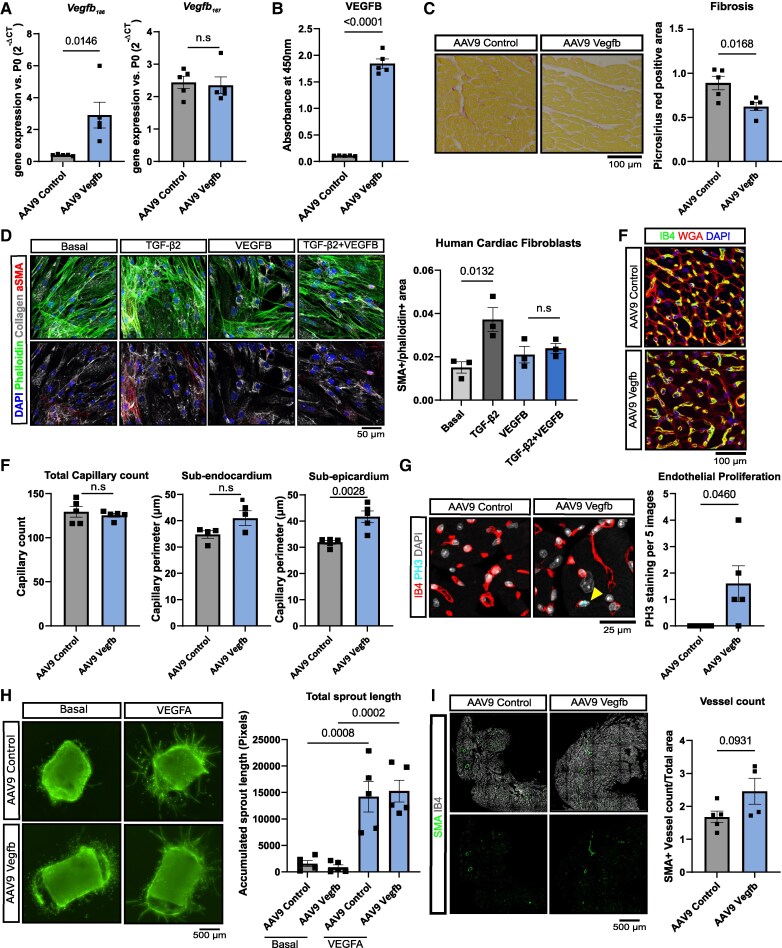
*Vegfb* overexpression attenuates fibrosis and denervation. (*A*, *B*) Eighteen-month-old mice were treated with AAV9 to overexpress Vegfb (AAV9 Vegfb). Empty vectors served as control (AAV9 Control). *Vegfb* induction was determined in heart samples by RT-qPCR against *Vegfb_186_* and *Vegfb_167_* (panel *A*, *n* = 5) and by ELISA (panel *B*, *n* = 5). (*C*) Picrosirius red staining in hearts of aged mice treated with AAV9-Vegfb vs. control (*n* = 5). (*D*) Human cardiac fibroblasts treated for 72 h with recombinant TGF-β2, VEGFB, and both in combination, immunostained with Phalloidin, Collagen 1a1, aSMA, and DAPI (*n* = 3). (*E*, *F*) Histological staining for Isolectin B4 (IB4, green), WGA, and DAPI in hearts of aged mice treated with AAV9-Vegfb vs. control (*n* = 5) to assess capillary count and perimeter. Panel (*F*) shows the quantification of capillary count and perimeter (μm) in the endocardium and epicardium (*n* = 5). (*G*) Assessment of endothelial proliferation (yellow arrow) using phosphor histone 3 (PH3), isolectin B4 (IB4), and DAPI in hearts of aged mice treated with AAV9-Vegfb vs. control (*n* = 5). (*H*) Angiogenic sprouting was determined by aortic ring assay applied on aortic rings from aged mice treated with AAV9-Vegfb vs. control (*n* = 5). Rings were cultured in the presence and absence of recombinant VEGFA. (*I*) Assessment of arteries using immunostaining with smooth muscle actin (SMA), Isolectin B4 (IB4) and DAPI in hearts of aged mice treated with AAV9-Vegfb vs. control (*n* = 5). Quantification is shown in the right part pf the panel as SMA + vessel count normalized to total tissue area. Data are shown as mean and error bars indicate SEM. Data are Gaussian distributed and *P*-value was assessed by two-tailed, unpaired Student’s *t*-test (*A*–*E* and *G*–*I*) or by using one-way ANOVA with a *post hoc* Tukey test (*F*).

To ask if restoring the available paracrine VEGFB pool in the aging heart can rescue age-related pathologies in the heart, we used adeno-associated virus vector 9 (AAV9) transduction for cardiac overexpression of *Vegfb_186_* in 18-month-old mice (hereafter referred to as AAV9-Vegfb). As a control, we used AAV9 vectors without a payload (hereafter referred to as AAV9-control). After 8 weeks of treatment, overexpression of *Vegfb_186_* was shown by quantitative RT-PCR with no discernible impact on the endogenous *Vegfb_167_* isoform, thus confirming a robust and sustained overexpression of the soluble *Vegfb* isoform (*Figure 2A*). ELISA analyses of the respective sera and immunoblotting of heart lysates further confirmed the induction of VEGFB_186_ protein expression by AAV9-Vegfb treatment (*Figure 2B*; Supplementary material online, *Figure S3*).

Next, we assessed the impact of *Vegfb* overexpression on the hallmarks of aging. Specifically, we determined its effect on age-related cardiac fibrosis and the vasculature. *Vegfb* overexpression significantly decreased fibrosis of the aging heart (*Figure 2C*; Supplementary material online, *Figure S4*). To investigate whether the attenuation of fibrosis could be a direct effect of VEGFB on fibroblasts, we cultured human cardiac fibroblasts with recombinant VEGFB. We found that recombinant VEGFB attenuated the TGF-β2-induced activation of cardiac fibroblasts, as demonstrated by reduced SMA staining (*Figure 2D*). This effect may depend on Neuropilin-1 (NRP1) that binds VEGFB and is expressed in human fibroblasts at both RNA^30^ and protein level, whereas VEGFR-1, the main VEGFB signal transducing tyrosine kinase receptor is not expressed (see Supplementary material online, *Figure S5*).

Since ageing reduces capillary density and increases capillary perimeter,^24,34^ we assessed these parameters histologically. Although the overall capillary density remained unchanged between AAV9-control and AAV9-Vegfb hearts, we found a significant increase in capillary perimeter in the sub-epicardial area in AAV9-Vegfb hearts (*Figure 2E and F*). As expected, we detected a mitogenic effect of VEGFB on endothelial cells, as evidenced by phospho-H3 staining (*Figure 2G*). To functionally assess the angiogenic effect of *Vegfb* overexpression, we used an *ex vivo* aortic ring assay using aortae from AAV9-control and AAV9-Vegfb treated aged mice. In line with the fact that VEGFB is a modest angiogenic factor, there was no increase in angiogenic sprouting upon *Vegfb* overexpression at baseline or when aortic ring outgrowth was additionally stimulated with recombinant VEGFA *in vitro* (*Figure 2H*). Furthermore, immunohistochemical staining for alpha smooth muscle actin (αSMA) did not indicate significant differences in vessel arterialization between the AAV9-control and AAV9-Vegfb treated mice (*Figure 2I*).

Together these data demonstrate that *Vegfb* overexpression attenuates cardiac fibrosis in aged mouse hearts. On microvascular level, *Vegfb* overexpression in aged hearts induced a mild mitogenic effect but increased capillary perimeter. In young mice, *Vegfb* overexpression induces cardiac endothelial proliferation that peaks at 2 weeks after gene delivery,^23^ which may explain the modest vascular response at the dose of the AAV vector we used at the 8-week time point.

### 3.3 *Vegfb* rescues ageing-mediated denervation

**Figure 3:**
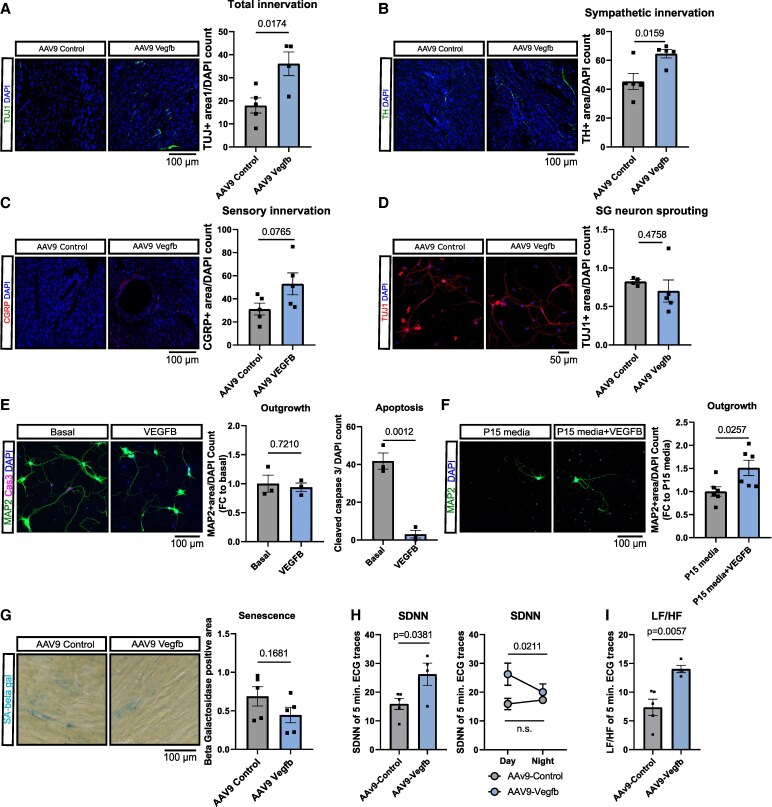
AAV9-mediated overexpression of *vegfb* rescues age-related cardiac denervation. (*A*–*C*) Histological assessment of left ventricular innervation in aged mice after AAV9-control vs. AAV9-Vegfb treatment. Axons were marked in green with TUJ1 (*A*, *n* = 5 vs. *n* = 4) or tyrosine hydroxylase-TH (*B*, *n* = 5). Sensory fibres were marked in red with calcitonin gene-related peptide—CGRP (*C*, *n* = 5). DAPI served as counterstain. (*D*) Neurons were isolated from the stellate ganglia (SG) from aged mice 8 weeks after AAV9-control vs. AAV9-Vegfb treatment. Neurons were cultured for 72 h and axon outgrowth was determined by TUJ1 and DAPI immunostaining (*n* = 4 vs. *n* = 5). (*E*) Mouse cortical neurons were cultured in the presence and absence of recombinant VEGFB. Axon outgrowth was determined by MAP-2 staining and apoptosis was determined by cleaved Caspase 3 staining (*n* = 3). (*F*) Mouse cortical neurons were cultured in supernatants from long-passaged (P15) human umbilical vein endothelial cells. The supernatant was supplemented with recombinant VEGFB. Axon outgrowth was determined by MAP-2 staining (*n* = 6). (*G*) Cardiac senescence was determined in hearts from aged mice after AAV9-Control vs. AAV9-Vegfb treatment (*n* = 5). (*H*, *I*) Heart rate variability was assessed via telemetric measurements in aged mice 6 weeks after AAV9-Control vs. AAV9-Vegfb treatment. Heart rate variability was assessed via time domain analysis (*H*, SDNN) and frequency domain (*I*, LF/HF) in *n* = 5 vs. *n* = 4 animals. Data are shown as mean and error bars indicate SEM. Data are Gaussian distributed and *P*-values were calculated using the two-tailed, unpaired Student’s *t*-test, a paired *t*-test was used to compare day and night cycles in panel (*H*).

We have previously reported that aging induces a denervation of the aging left ventricle.^24^ Since VEGFB has neuroprotective effects and has been implicated in peripheral neuron regeneration,^22,35^ we hypothesized that *Vegfb* overexpression could rescue age-induce cardiac denervation. Indeed, we observed an overall increase in left ventricular innervation following AAV9-Vegfb treatment in cardiac sections stained for the pan-neuronal marker TUJ1 (*Figure 3A*). To specify which type of nervous fibres are rescued, we histologically assessed sympathetic fibres by tyrosine hydroxylase and sensory fibres by calcitonin-gene-related peptide. The sympathetic fibre density was significantly increased while sensory fibre density tended to be increased in hearts of aged AAV9-Vegfb treated mice (*Figure 3B and C*). The parasympathetic marker acetylcholine esterase, however, was not changed upon *Vegfb* overexpression (see Supplementary material online, *Figure S6*).

To gain insights into the mechanisms underlying the VEGFB-induced re-innervation of the heart, we investigated whether AAV9-Vegfb treatment directly induces neuron and axon growth. Hence, we isolated neurons from the sympathetic source of the heart, the stellate ganglia. The stellate ganglia are paired sympathetic ganglion structures that reside bilaterally in the first intercostal space.^25^ They project towards the heart and innervate the atria and ventricles. We isolated the stellate ganglia from AAV9-control vs. AAV9-Vegfb treated aged mice and purified the respective neurons. After seeding the cells, we did not find differences in axon outgrowth between neurons from AAV9-control vs. AAV9-Vegfb treated mice (*Figure 3D*). Interestingly, culturing neurons in the presence of recombinant VEGFB did not induce axon sprouting, but reduced cleaved caspase 3 (*Figure 3E*). This suggests that VEGFB does not induce axon sprouting but protects neurons from apoptosis. Since vascular senescence contributes to age-related cardiac denervation *in vivo*,^24^ we additionally assessed whether recombinant VEGFB rescues axon outgrowth in the presence of conditioned media derived from senescent cells. Indeed, VEGFB treatment rescued the reduced neurite outgrowth induced by conditioned media derived from senescent (late passage) endothelial cells (*Figure 3F*). In addition, we investigated whether AAV9-Vegfb affects senescence *in vivo*. Although we observed a trend towards decreased acidic β-galactosidase staining, this difference did not reach statistical significance (*Figure 3G*).

To finally evaluate whether *Vegfb* overexpression functionally rescues sympathetic innervation in the aging heart, we remotely recorded ECG traces from aged AAV9-control and AAV9-Vegfb treated mice using telemetry. This enabled us to assess heart rate variability *in vivo*, a key indicator of sympathetic influence on cardiac function, which is decreased in the elderly.^24^ Heart rate variability can be determined by both time and frequency domain measurements. Time domain analysis following six weeks of *Vegfb* induction showed an increased standard deviation of the intervals between heartbeats (SDNN) in the *Vegfb* group during daytime. In addition, VEGFB significantly restored the SDNN day-night-rhythm in aged mice (*Figure 3H*). Consistently, frequency domain analysis revealed an increased LF/HF ratio during daytime as well, indicative of heightened sympathetic activity, further indicating enhanced sympathetic modulation (*Figure 3I*). The elevation of both parameters confirms an increase in heart rate variability upon *Vegfb* transduction in aged mice.

Taken together, these data suggest that VEGFB exerts a neuroprotective effect, restoring cardiac innervation in the aging heart, which is functionally linked to an increase in heart rate variability. However, VEGFB did not significantly influence cellular senescence in the aging heart.

### 3.4 *Vegfb* overexpression rescues age-induced diastolic dysfunction but increases cardiac mass

**Figure 4:**
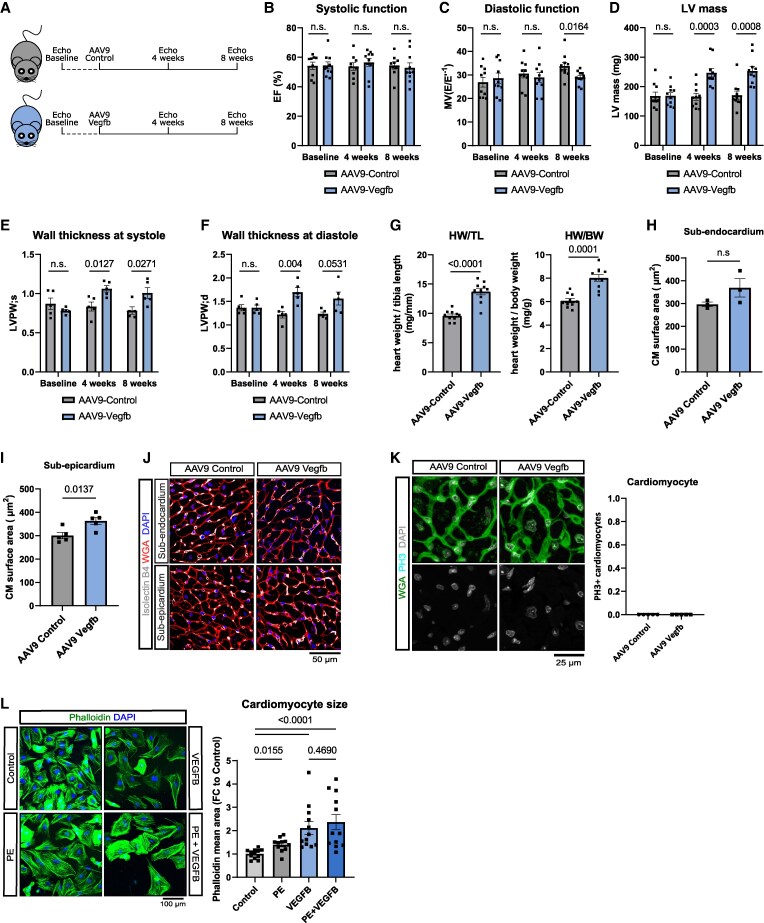
AAV9-mediated overexpression of *vegfb* delays age-related diastolic dysfunction but induces cardiac hypertrophy in aging heart. (*A*) Schematic representation showing the experimental design. (*B*–*F*) Echocardiography of 18-month-old mice before (baseline) and 4 and 8 weeks after AAV9-control/AAV9-Vegfb injection (*n* = 10). (*B*) Systolic function represented as ejection fraction [EF (%)]. (*C*) Diastolic function represented as MV (E/E′^−1^). (*D*) Left ventricular mass. (*E*, *F*) Left ventricular wall thickness at systole and diastole (*n* = 5). (*G*) Heart weight as determined by heart weight vs. tibia length or body weight in aged mice 8 weeks after AAV9 injection (*n* = 10). (*H*–*J*) Histological assessment of cardiac hypertrophy in the sub-endocardial and sub-epicardial area by WGA staining. IB4 and DAPI served as counter stain (*n* = 5). (*K*) Immunostaining of the cardiomyocytes against phosphor histone 3 (PH3), WGA, and DAPI (*n* = 5). (*L*) Neonatal rat cardiomyocytes cultured in the presence and absence of phenylephrine (PE), recombinant VEGFB, and PE + VEGFB (*n* = 3). Data are shown as mean and error bars indicate SEM. Data are Gaussian distributed and *P*-value was assessed by two-tailed, unpaired Student’s *t*-test (*B–K*) or by using one-way ANOVA with a *post hoc* Tukey test (*L*).

To determine the overall functional impact of the observed VEGFB-mediated alterations, we conducted echocardiography on both experimental groups before administrating the AAV9 vectors (baseline) and at 4 and 8 weeks post-delivery (*Figure 4A*). Notably, we observed a stable systolic function in both groups as indicated by preserved ejection fraction throughout the entire observation period (*Figure 4B*). However, a notable difference emerged concerning diastolic function, which was preserved after 8 weeks in the AAV9-Vegfb group, while a significant decline was detected in the AAV9-control group (*Figure 4C*). We also noted an elevation in left ventricular mass in the AAV9-Vegfb group, accompanied by an increase in left ventricular wall thickness at both systole and diastole (*Figure 4D–F*). In line with this, heart weight to tibia length and heart weight to body weight ratios were both elevated in the AAV9-Vegfb treated mice (*Figure 4G*).

The increase in cardiac mass can be mediated by induction of cardiomyocyte hypertrophy or cardiomyocyte proliferation. The AAV9-Vegfb treated mice, indeed, showed increased hypertrophy predominantly in the sub-epicardial area but only a trend in the sub-endocardial area (*Figure 4H–J*). In contrast, no induction in cardiomyocyte proliferation was observed following *Vegfb* overexpression, as indicated by phospho-histone 3 staining of mitotic cells (*Figure 4K*). To further substantiate the hypertrophy-inducing potential of VEGFB, we cultured isolated neonatal rat cardiomyocytes in the presence and absence of recombinant VEGFB. Consistent with our *in vivo* observations, recombinant VEGFB-induced cardiomyocyte hypertrophy in the presence and absence of phenylephrine (*Figure 4L*). Together these data suggest that *Vegfb* overexpression improves diastolic heart function but further augments hypertrophy of the aging heart.

### 3.5 Transcriptomic analysis reveals possible involvement of STAT3 in VEGFB-induced cardiac hypertrophy

**Figure 5:**
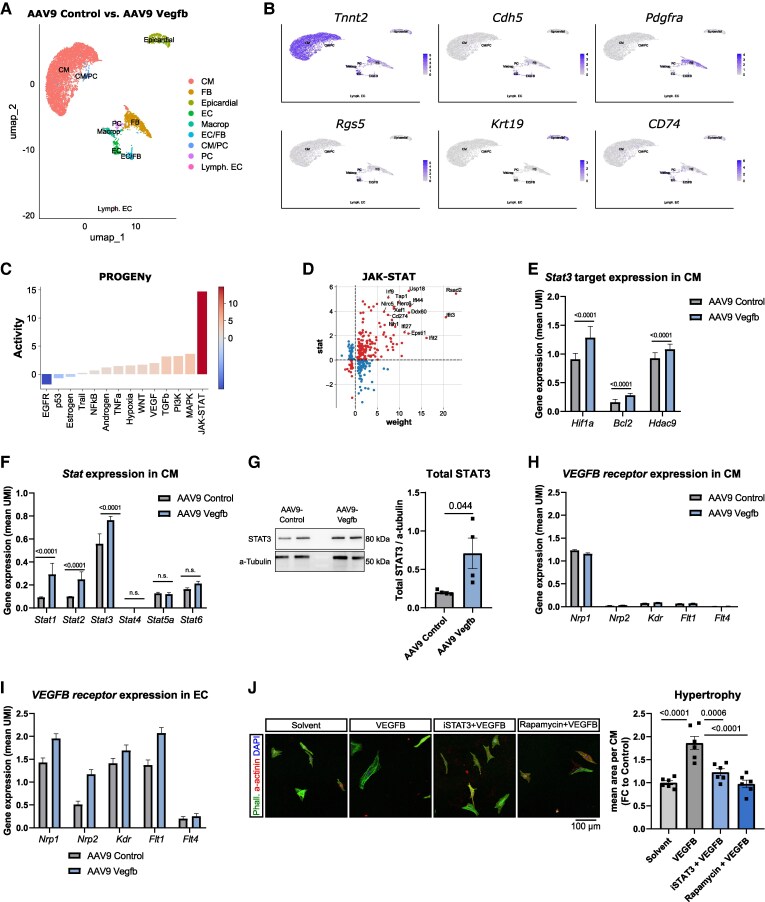
STAT3 contributes to VEGFB-induced cardiac hypertrophy. Single-nuclei RNA sequencing of hearts from aged mice after 8 weeks after AAV9-Control vs. AAV9-Vegbf injection (*n* = 3). (*A*) UMAP plot representing the distinct cell types of the heart in all conditions. (*B*) UMAP plots showing different cell type markers for cardiomyocytes (*Tnnt2*), endothelial cells (*Cdh5*), fibroblasts (*Pdgfra*), immune cells (*CD74*), epicardial cells (*Krt19*), and pericytes (*Rgs5*). (*C*) PROGENy pathway analysis of differentially regulated genes in the cardiomyocyte of AAV9-Vegfb vs. AAV9-control treated aged mice. (*D*) Dot plot displaying JAK-STAT-related genes that are dysregulated in the cardiomyocyte cluster. (*E*, *F*) STAT3 target expression (*E*) and STAT family expression levels (*F*) displayed as mean UMI in the cardiomyocyte cluster of AAV9-Vegfb vs. AAV9-Control treated aged mice (*n* = 3). (*G*) Western blot of phosphor-STAT3 and total STAT3 vs. a-tubulin in whole hearts of AAV9-Vegfb vs. AAV9-Control treated aged mice (*n* = 5). (*H*, *I*) VEGF receptor expression levels displayed as mean UMI in the cardiomyocyte (*H*) and endothelial cells cluster (*I*) of AAV9-Vegfb vs. AAV9-Control treated aged mice (*n* = 3). (*J*) Isolated neonatal rat cardiomyocytes were cultured for 72 h in the presence and absence of recombinant mouse VEGFB and in VEGFB + STAT3 inhibitor (iSTAT3) or rapamycin (*n* = 6). Cardiomyocyte hypertrophy was measured as α-actinin area per nucleus (DAPI). *P*-value was calculated using a Wilcox-test (*E*, *F*, *H*, and *I*) or the One-way ANOVA with a *post hoc* Tukey test (*H*).

To gain further insight into the molecular signatures involved in AAV9-Vegfb driven cardiac hypertrophy, we performed snRNA sequencing on snap-frozen heart tissues obtained from three AAV9-control vs. three AAV9-Vegfb treated 20-month-old mice, 8 weeks after AAV9-injection (*Figure 5A*). The use of marker genes such as *Tnnt2*, *Cdh5*, *Pdgfra*, *Rgs5, Krt19,* and *Cd74* allowed us to identify the respective cell clusters, such as cardiomyocytes, endothelial cells, fibroblasts, pericytes, epicardial cells, and immune cells, respectively (*Figure 5B*). This analysis confirmed the expression of the *Vegfb* specifically in cardiomyocytes (see Supplementary material online, *Figure S7*). To explore the impact of *Vegfb* overexpression on cardiomyocytes, we determined differentially regulated genes (see Supplementary material online, *Figure S8*). We also used the PROGENy pathway analysis,^31^ which predicts possible signalling pathways by taking the regulation of putative downstream targets into account. Interestingly, *Vegfb* overexpression was associated with increased expression of transcripts involved in the JAK-STAT pathway and its downstream targets in cardiomyocytes (*Figure 5C* and *D*). KEGG cluster pathway analysis confirmed the regulation STAT pathways (see Supplementary material online, *Figure S9*) and the regulation of its respective target genes (see Supplementary material online, *Table S1*), such as *Hif1a*, *Bcl2,* and *Hdac9* (*Figure 5E*). Interestingly, *Vegfb* overexpression increased the expression of *Stat1*, *Stat2,* and *Stat3*, but not *Stat4*, *Stat5a,* and *Stat6* in cardiomyocytes (*Figure 5F*). Of these, *Stat3* showed the highest expression level, thus we confirmed its induction at protein level in whole heart lysates from aged mice after 8 weeks of AAV9-Vegfb treatment (*Figure 5G*; Supplementary material online, *Figure S10*). These data suggested the involvement of STAT3 in VEGFB-driven cardiac hypertrophy, although snRNA seq data from human or mouse hearts indicated that only VEGFB co-receptor *NRP1*/*Nrp1* was expressed in cardiomyocytes, but not *Nrp2*, VEGFR1 (*Flt1*), VEGFR2 (*Kdr*), and VEGFR3 (*Flt4*) (see Supplementary material online, *Figure S5*; *Figure 5H and I*).

To investigate if VEGFB may induce cardiomyocyte hypertrophy via STAT3, we studied the effect of VEGFB on cardiomyocytes *in vitro*. We cultured neonatal rat cardiomyocytes, that express VEGFB receptors at levels 3 orders of magnitude less than cultured human endothelial cells^36^ were cultured in the presence and absence of recombinant VEGFB with and without STAT3 inhibitor (STAT3 inhibitor XIII). Besides using the STAT3 inhibitor, cardiomyocytes were treated with rapamycin to block mTOR as a positive control for anabolic biosynthetic pathways. Interestingly, both the STAT3 inhibitor and rapamycin inhibited the VEGFB-mediated cardiomyocyte hypertrophy (*Figure 5J*).

These data suggest that VEGFB may stimulate cardiomyocyte hypertrophy in part via STAT3; yet additional studies will be needed to understand the precise upstream and downstream pathways.

### 3.6 VEGFB induces physiological cardiac hypertrophy in aged mice

Cardiac hypertrophy is considered as an adaptive response to hemodynamic stress, with physiological hypertrophy being a compensatory mechanism to enhance cardiac performance. However, pathological hypertrophy can lead to myocardial dysfunction, fibrosis, and an increased risk of adverse events such as arrhythmias, myocardial infarction, and sudden death. There is evidence that VEGFB induces physiological-like cardiac hypertrophy.^23,37^ However, in pathological conditions, VEGFB overexpression in hypertensive rats resulted in pathological hypertrophy and a shift from diastolic to systolic heart failure.^38^ To clarify whether VEGFB induces physiological or pathological cardiac hypertrophy in aged mice, we assessed specific hypertrophy markers by histological and transcriptomic analysis.

**Figure 6:**
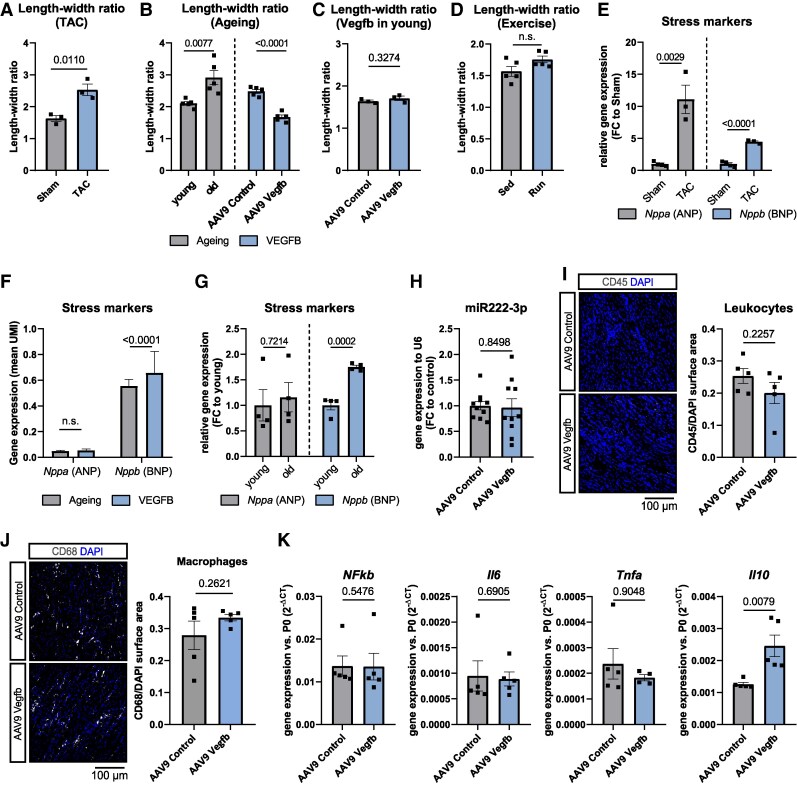
VEGFB induces cardiac hypertrophy in aged mice. (*A*) Length-width ratio of cardiomyocytes after TAC surgery (*n* = 3). (*B*) Shown is the length-width ratio of cardiomyocytes from young vs. aged mice (*n* = 5) and from aged mice 2 months after AAV9-control or AAV9-Vegfb treatment (*n* = 5). (*C*) Length-width ratio of cardiomyocytes from young mice with and without AAV9-Vegfb treatment (*n* = 3, 4 weeks of treatment). (*D*) Length-width ratio of cardiomyocytes from young mice after 2 months of voluntary exercise (*n* = 5). (*E*–*G*) Expression of *Nppa* and *Nppb* in sham vs. TAC-operated mouse hearts (*E*, *n* = 3), in hearts from aged AAV9-control vs. AAV9-Vegfb treated mice (*F*, *n* = 3) and in young vs. old whole mouse tissue (*G*, *n* = 4). (*H*) Expression of miR-222-3p in hearts from aged AAV9-Control vs. AAV9-Vegfb treated mice (*n* = 10). (*I*, *J*) Immunolabelling of CD45 + leucocytes (*I*) and CD68 + macrophages (*J*) in hearts from aged mice 2 month after AAV9-control or AAV9-Vegfb treatment (*n* = 5). (*K*) Comparison of *Nfkb*, *Il6*, *Tnfa*, *Il10* transcript levels in hearts from aged AAV9-control vs. AAV9-Vegfb treated mice (*n* = 5). *P*-values were calculated using two-sided *t*-test (*A–J*), Mann–Whitney test (*K*) or Wilcox-test (*F*).

One suitable indicator to discriminate between pathological and physiological hypertrophy is the cardiomyocyte length-width ratio.^39^ Physiological hypertrophy is characterized by symmetric increase in both cardiomyocyte length and width, without altering the length-width ratio. In contrast, pathological hypertrophy leads to an altered cardiomyocyte length-width ratio.^40^ To determine the respective type of hypertrophy histologically, we assessed WGA-stained heart sections from young and aged mice. As a positive control for pathological hypertrophy, we used heart sections from mice after trans-aortic constriction,^41^ in which the length-width ratio was increased (*Figure 6A*). Aged mouse hearts had an elevated length-width ratio (*Figure 6B*). Interestingly, after AAV9-Vegfb treatment, the aged mice showed a decrease in the cardiomyocyte length-width ratio, suggesting that VEGFB can rescue the age-related increase in cardiomyocyte length-width ratio (*Figure 6B*). Heart sections from young mice after AAV9-Vegfb treatment or young mice after 8 weeks of volunteer exercise also developed hypertrophy,^42^ without a change in the length-width ratio (see Supplementary material online, *Figure S11* and *Figure 6C* and *D*). These data suggest that VEGFB induces protective cardiac hypertrophy in aged mice by rescuing the age-related increase in length-width ratio.

Apart from histological changes, pathological hypertrophy is characterized by specific biomarkers such as *Nppa* and *Nppb* (encoding atrial and brain natriuretic factor/peptide, respectively). Whereas, TAC induced the expression of *Nppa* and *Nppb* by 11.1− and 4.5-fold, respectively (sequencing data^43^) AAV9-Vegfb treatment increased only *Nppb* by 1.2-fold. Mouse ageing by itself also induced only *Nppb,* by 1.8-fold^28^ (*Figure 6E–G*). Also miR-222-3p expression, a marker of pathological hypertrophy^44^ was unchanged by AAV9-Vegfb treatment (*Figure 6H*).

Pathological hypertrophy is characterized by cardiac dysfunction, fibrosis and inflammation.^45^ As described above, *Vegfb* overexpression in aged mice improved diastolic function (*Figure 4C*) and led to decreased cardiac fibrosis (*Figure 2C*). To assess cardiac inflammation, we quantified CD45 + leucocytes and CD68 + macrophages and the pro-inflammatory markers *Nfkb*, *Il6*, and *Tnfa* in the hearts of aged mice after AAV9-control vs. AAV9-Vegfb treatment, but did not observe any differences between the groups (*Figure 6I–K*). In contrast, the anti-inflammatory marker *Il10* was even up-regulated upon *Vegfb* overexpression (*Figure 6K*). Our snRNA seq data revealed no changes in selective inflammatory markers (*Tnf*, *Il6*, *Il1b*, *Tgfb1*, and *Tgfb2*) in any of the individual cell clusters, as well. Furthermore, expression of the profibrotic *Tgfb2* transcript was repressed in lymphatic endothelial cells and macrophages (see Supplementary material online, *Figure S12*). In summary, our results suggest that in aged hearts, VEGFB can induce compensatory hypertrophy, improved diastolic function, and reduced cardiac fibrosis, while inflammation was not significantly affected.

## 4. Discussion

In our current study, we noted a decline in *Vegfb_186_* levels in the aging mouse heart, particularly in endothelial cells and cardiomyocytes. When we counteracted the age-related decline of *Vegfb* expression by introducing *Vegfb_186_* through AAV9-mediated overexpression in aged mice, we observed that the age-related diastolic dysfunction was alleviated, while systolic function remained unchanged. Furthermore, AAV9-Vegfb treatment led to a reduction in cardiac fibrosis and an improvement in cardiac innervation at doses which had only a modest effect on cardiac vessels. However, *Vegfb* overexpression induced cardiac hypertrophy also in aged mice and cardiac snRNA sequencing suggested that STAT3 might be involved in the VEGFB-mediated cardiomyocyte hypertrophy. Further experiments are needed to dissect the precise molecular mechanisms of VEGFB-induced hypertrophy, especially to elucidate a potential direct effect of VEGFB on cardiomyocytes.

The observed decline in *Vegfb_186_* expression in aging and diseased heart raises intriguing possibilities regarding the protective role of VEGFB. Indeed, accumulating evidence underlines the potential therapeutic significance of VEGFB, as its overexpression has been shown to induce metabolic reprogramming and preserve cardiac function in models of cardiac injury.^17,18^ In line with this, our study unveiled protection from age-related diastolic dysfunction in aged mice following AAV9-Vegfb treatment. However, it is crucial to acknowledge the complex nature of the effects of VEGFB on the heart. While VEGFB demonstrates its ability in rescue cardiac function, it concurrently triggers cardiac hypertrophy, a finding previously observed in young adult and diseased mice.^20,23,36^ To assess whether VEGFB induces physiological or pathological cardiac hypertrophy in aged mice, we investigated the cardiomyocyte length-to-width ratio, which reflects a suitable measure to discriminate between physiological and pathological hypertrophy. We observed that ageing leads to an increase of the cardiac length-to-width ratio, which is reversed in aged mice after AAV9-Vegfb treatment. In fact, the length-to-width ratio in aged AAV9-Vegfb-treated mice was comparable to that in young wildtype mice. Most interestingly, AAV9-Vegfb treatment did induce hypertrophy in young mice without changing the length-to-width ratio. These data suggest that at histological level, VEGFB does induce physiological hypertrophy in aged mice by decreasing the age-related increase in cardiac length-to-width ratio. Pathological hypertrophy is characterized by functional decline, cardiac fibrosis, and inflammation.^45^ Here, aged mice after AAV9-Vegfb-treatment displayed improved diastolic function and heart rate variability, accompanied by reduced fibrosis and unaffected inflammatory marker expression. These data highlight the beneficial effect of VEGFB on the ageing heart and suggest that VEGFB overall induces cardioprotective effects in aged mice.

Beyond its impact on cardiomyocytes, VEGFB may influence vascular function, potentially leading to improvements in overall heart function. Previous studies have highlighted a role of VEGFB in promoting coronary vessel formation without necessarily triggering sprouting angiogenesis, thereby contributing to cardiac regeneration following various insults in adult mice.^14,15^ In line with these findings, our experiments with AAV9-Vegfb treatment did not induce angiogenic sprouting in aortic rings from aged mice. However, we were unable to discern significant effects on cardiac arteries or arterioles in histological analysis. Cardiac hypertrophy often leads to a relative decrease in capillary density,^46^ which however was not changed in aged mice after *Vegfb* overexpression despite sustainable cardiac hypertrophy. The observation that AAV9-Vegfb treatment had a mild mitotic effect on cardiac endothelial cells suggests that VEGFB induces endothelial proliferation and thereby protects from cardiac hypertrophy related capillary density decline. Recent studies which analysed endothelial cell proliferation after *Vegfb* overexpression in young mice revealed that endothelial proliferation peaked at 2 weeks after *Vegfb* overexpression and declined afterwards.^23^ Hence, it is likely that we missed the peak proliferation in endothelial cells by inspecting the hearts sections 8 weeks after *Vegfb* overexpression.

Diastolic dysfunction is a prominent feature of cardiac aging, characterized by impaired ventricular relaxation and increased ventricular stiffness.^9^ Given that cardiac aging is closely linked to fibrosis^9,10^ and that fibrosis is associated with diastolic dysfunction,^47,48^ we conducted an assessment of fibrosis in the hearts of both experimental groups. Remarkably, the hearts of aged mice treated with AAV9-Vegfb exhibited a significantly lower degree of diffuse fibrosis compared to the control group. Such reduction in fibrosis could lead to decreased ventricular stiffness and delay the onset of age-related diastolic dysfunction. The observed anti-fibrotic effect in response to AAV9-Vegfb treatment may be mediated via *Nrp1* expressed in the cardiac fibroblasts.

Furthermore, we observed a restoration of left ventricular axon density in the hearts of aged mice following AAV9-Vegfb treatment. Previous findings from our own research have indicated a dysregulation of the neurotrophic factor Semaphorin-3A in the endothelium of aged mouse hearts, resulting in reduced left ventricular innervation.^24^ Our present data align with this notion, suggesting that VEGFB may function as a neuroprotective factor in aging hearts. However, unlike Semaphorin-3A, which directly influences neurite outgrowth, VEGFB did not directly alter neurite sprouting. Instead, VEGFB seemed to have a neuroprotective function by mitigating the anti-neurite-sprouting and pro-apoptotic effects induced by senescent endothelial cell supernatants. In this regard it is noteworthy to mention that both Semaphorin-3A and VEGFB have the ability to bind to NRP-1.^49^ The up-regulation of Semaphorin-3A in aged cardiac endothelial cells^24^ and the observed decline in *Vegfb* expression might lead to a shift in neuropilin-1 signalling, resulting in axon regression. One limitation in our study is that the precise molecular mechanism underlying VEGFB-induced cardiac re-innervation was not addressed. This knowledge gap should be covered in future studies.

VEGFB has been considered as a neuroprotective factor in the context of cardiac stress.^21^ However, high levels of *Vegfb_186_* transgene expression lead to cardiac hypertrophy that can make the heart more susceptible to dobutamine-induced arrhythmias and sudden cardiac death.^21^ Thus one caveat is that excessive *Vegfb_186_* overexpression could potentially lead to uncontrolled nerve fibre patterning and subsequent arrhythmias, yet this concern is attenuated by the normal lifespan of transgenic mice and rats expressing high levels of the endogenous Vegfb gene in cardiomyocytes, under the alpha-myosin heavy chain promoter.^15^

However, it is worth considering that defining the specific site and dosage of *Vegfb_186_* induction in the heart might be a valuable strategy to minimize unintended off-target effects. Given that *Vegfb* is moderately repressed in aged cardiac endothelial cells and cardiomyocytes, it may be interesting to explore, whether a titrated manipulation of VEGFB in aged hearts could lead to an optimal therapeutic approach. Understanding the signalling pathway underlying VEGFB-induced re-innervation may provide direct targets to modulate cardiac innervation without inducing VEGFB expression.

Translational perspective This study highlights the promising potential of VEGFB gene therapy as an innovative strategy to combat age-related cardiac decline. By demonstrating that VEGFB overexpression enhances cardiac function, reduces fibrosis, and improved sympathetic innervation in the hearts of aged mice, we lay the groundwork for novel therapeutic approaches targeting age-associated heart conditions. The observed benefits—including improved diastolic function and enhanced heart rate variability—suggest that VEGFB-based therapies could meaningfully improve the quality of life for elderly patients with cardiac issues. Moving forward, clinical trials will be essential to confirm these findings in humans and establish VEGFB gene therapy as a safe, effective intervention for age-related cardiac dysfunction.

## Supplementary Material

cvaf046_Supplementary_Data

## Data Availability

All data are available in the manuscript or the supplementary materials. The single cell experiment is available at Arrayexpress under the accession E-MTAB-14595. Material is available upon request from the authors indicated in the supplementary material.

## References

[1] Benjamin EJ, Blaha MJ, Chiuve SE, Cushman M, Das SR, Deo R, de Ferranti SD, Floyd J, Fornage M, Gillespie C, Isasi CR, Jiménez MC, Jordan LC, Judd SE, Lackland D, Lichtman JH, Lisabeth L, Liu S, Longenecker CT, Mackey RH, Matsushita K, Mozaffarian D, Mussolino ME, Nasir K, Neumar RW, Palaniappan L, Pandey DK, Thiagarajan RR, Reeves MJ, Ritchey M, Rodriguez CJ, Roth GA, Rosamond WD, Sasson C, Towfighi A, Tsao CW, Turner MB, Virani SS, Voeks JH, Willey JZ, Wilkins JT, Wu JHY, Alger HM, Wong SS, Muntner P. Heart disease and stroke statistics—2017 update: a report from the American Heart Association. Circulation 2017;135:e146–e603. 28122885 10.1161/CIR.0000000000000485 PMC5408160

[2] Dimmeler S . Regulation of bone marrow-derived vascular progenitor cell mobilization and maintenance. Arterioscler Thromb Vasc Biol 2010;30:1088–1093. 20453167 10.1161/ATVBAHA.109.191668

[3] Ding B, Nolan DJ, Guo P, Babazadeh AO, Cao Z, Rosenwaks Z, Crystal RG, Simons M, Sato TN, Worgall S, Shido K, Rabbany SY, Rafii S. Endothelial-derived inductive angiocrine signals initiate and sustain regenerative lung alveolarization. Cell 2012;147:539–553. 10.1016/j.cell.2011.10.003 PMC3228268 22036563

[4] Ding B-S, Cao Z, Lis R, Nolan DJ, Guo P, Simons M, Penfold ME, Shido K, Rabbany SY, Rafii S. Divergent angiocrine signals from vascular niche balance liver regeneration and fibrosis. Nature 2014;505:97–102. 24256728 10.1038/nature12681 PMC4142699

[5] Hu J, Srivastava K, Wieland M, Runge A, Mogler C, Besemfelder E, Terhardt D, Vogel MJ, Cao L, Korn C, Bartels S, Thomas M, Augustin HG. Endothelial cell-derived angiopoietin-2 controls liver regeneration as a spatiotemporal rheostat. Science (1979) 2014;343:416–419. Available from: http://science.sciencemag.org/content/343/6169/416.abstract. 10.1126/science.1244880 24458641

[6] Manavski Y, Boon RA, Dimmeler S. Vascular niche controls organ regeneration. Circ Res 2014;114:1077–1079. 24677233 10.1161/CIRCRESAHA.114.303452

[7] Bassat E, Mutlak YE, Genzelinakh A, Shadrin IY, Baruch Umansky K, Yifa O, Kain D, Rajchman D, Leach J, Riabov Bassat D, Udi Y, Sarig R, Sagi I, Martin JF, Bursac N, Cohen S, Tzahor E. The extracellular matrix protein agrin promotes heart regeneration in mice. Nature 2017;547:179–184. 28581497 10.1038/nature22978 PMC5769930

[8] Rhee S, Paik DT, Yang JY, Nagelberg D, Williams I, Tian L, Roth R, Chandy M, Ban J, Belbachir N, Kim S, Zhang H, Phansalkar R, Wong KM, King DA, Valdez C, Winn VD, Morrison AJ, Wu JC, Red-Horse K. Endocardial/endothelial angiocrines regulate cardiomyocyte development and maturation and induce features of ventricular non-compaction. Eur Heart J 2021;42:4264–4276. 34279605 10.1093/eurheartj/ehab298 PMC8560211

[9] Obas V, Vasan RS. The aging heart. Clin Sci 2018;132:1367–1382. 10.1042/CS20171156 29986877

[10] Wagner JUG, Dimmeler S. Cellular cross-talks in the diseased and aging heart. J Mol Cell Cardiol 2020;138:136–146. 31783034 10.1016/j.yjmcc.2019.11.152

[11] Cao Y . Positive and negative modulation of angiogenesis by VEGFR1 ligands. Sci Signal 2009;2:re1. 19244214 10.1126/scisignal.259re1

[12] Alitalo K, Tammela T, Petrova TV. Lymphangiogenesis in development and human disease. Nature 2005;438:946–953. 16355212 10.1038/nature04480

[13] Grunewald M, Kumar S, Sharife H, Volinsky E, Gileles-Hillel A, Licht T, Permyakova A, Hinden L, Azar S, Friedmann Y. Counteracting age-related VEGF signaling insufficiency promotes healthy aging and extends life span. Science 2021;373:eabc8479. 34326210 10.1126/science.abc8479

[14] Bry M, Kivelä R, Holopainen T, Anisimov A, Tammela T, Soronen J, Silvola J, Saraste A, Jeltsch M, Korpisalo P, Carmeliet P, Lemström KB, Shibuya M, Ylä-Herttuala S, Alhonen L, Mervaala E, Andersson LC, Knuuti J, Alitalo K. Vascular endothelial growth factor-B acts as a coronary growth factor in transgenic rats without inducing angiogenesis, vascular leak, or inflammation. Circulation 2010;122:1725–1733. 20937974 10.1161/CIRCULATIONAHA.110.957332

[15] Räsänen M, Sultan I, Paech J, Hemanthakumar KA, Yu W, He L, Tang J, Sun Y, Hlushchuk R, Huan X, Armstrong E, Khoma O-Z, Mervaala E, Djonov V, Betsholtz C, Zhou B, Kivelä R, Alitalo K. VEGF-B Promotes endocardium-derived coronary vessel development and cardiac regeneration. Circulation 2021;143:65–77. 33203221 10.1161/CIRCULATIONAHA.120.050635

[16] Räsänen M, Degerman J, Nissinen TA, Miinalainen I, Kerkelä R, Siltanen A, Backman JT, Mervaala E, Hulmi JJ, Kivelä R, Alitalo K. VEGF-B gene therapy inhibits doxorubicin-induced cardiotoxicity by endothelial protection. Proc Natl Acad Sci U S A 2016;113:13144–13149. 27799559 10.1073/pnas.1616168113 PMC5135329

[17] Kivelä R, Bry M, Robciuc MR, Räsänen M, Taavitsainen M, Silvola JMU, Saraste A, Hulmi JJ, Anisimov A, Mäyränpää MI, Lindeman JH, Eklund L, Hellberg S, Hlushchuk R, Zhuang ZW, Simons M, Djonov V, Knuuti J, Mervaala E, Alitalo K. VEGF-B-induced vascular growth leads to metabolic reprogramming and ischemia resistance in the heart. EMBO Mol Med 2014;6:307–321. 24448490 10.1002/emmm.201303147 PMC3958306

[18] Shang R, Lal N, Lee CS, Zhai Y, Puri K, Seira O, Boushel RC, Sultan I, Räsänen M, Alitalo K, Hussein B, Rodrigues B. Cardiac-specific VEGFB overexpression reduces lipoprotein lipase activity and improves insulin action in rat heart. Am J Physiol Endocrinol Metab 2021;321:E753–E765. 34747201 10.1152/ajpendo.00219.2021

[19] Nicin L, Schroeter SM, Glaser SF, Schulze-Brüning R, Pham M-D, Hille SS, Yekelchyk M, Kattih B, Abplanalp WT, Tombor L, Müller OJ, Braun T, Meder B, Reich C, Arsalan M, Holubec T, Walther T, Emrich F, Krishnan J, Zeiher AM, John D, Dimmeler S. A human cell atlas of the pressure-induced hypertrophic heart. Nat Cardiovasc Res 2022;1:174–185. 39195989 10.1038/s44161-022-00019-7 PMC11357985

[20] Huusko J, Lottonen L, Merentie M, Gurzeler E, Anisimov A, Miyanohara A, Alitalo K, Tavi P, Ylä-Herttuala S. AAV9-mediated VEGF-B gene transfer improves systolic function in progressive left ventricular hypertrophy. Mol Ther 2012;20:2212–2221. 23089731 10.1038/mt.2012.145 PMC3519981

[21] Lähteenvuo J, Hätinen O-P, Kuivanen A, Huusko J, Paananen J, Lähteenvuo M, Nurro J, Hedman M, Hartikainen J, Laham-Karam N, Mäkinen P, Räsänen M, Alitalo K, Rosenzweig A, Ylä-Herttuala S. Susceptibility to cardiac arrhythmias and sympathetic nerve growth in VEGF-B overexpressing myocardium. Mol Ther 2020;28:1731–1740. 32243833 10.1016/j.ymthe.2020.03.011 PMC7335717

[22] Guaiquil VH, Pan Z, Karagianni N, Fukuoka S, Alegre G, Rosenblatt MI. VEGF-B selectively regenerates injured peripheral neurons and restores sensory and trophic functions. Proc Natl Acad Sci U S A 2014;111:17272–17277. 25404333 10.1073/pnas.1407227111 PMC4260560

[23] Sultan I, Ramste M, Peletier P, Hemanthakumar KA, Ramanujam D, Tirronen A, von Wright Y, Antila S, Saharinen P, Eklund L, Mervaala E, Ylä-Herttuala S, Engelhardt S, Kivelä R, Alitalo K. Contribution of VEGF-B-induced endocardial endothelial cell lineage in physiological versus pathological cardiac hypertrophy. Circ Res 2024;134:1465–1482. 38655691 10.1161/CIRCRESAHA.123.324136 PMC11542978

[24] Wagner JUG, Tombor LS, Malacarne PF, Kettenhausen L-M, Panthel J, Kujundzic H, Manickam N, Schmitz K, Cipca M, Stilz KA, Fischer A, Muhly-Reinholz M, Abplanalp WT, John D, Mohanta SK, Weber C, Habenicht AJR, Buchmann GK, Angendohr S, Amin E, Scherschel K, Klöcker N, Kelm M, Schüttler D, Clauss S, Günther S, Boettger T, Braun T, Bär C, Pham M-D, Krishnan J, Hille S, Müller OJ, Bozoglu T, Kupatt C, Nardini E, Osmanagic-Myers S, Meyer C, Zeiher AM, Brandes RP, Luxán G, Dimmeler S. Aging impairs the neurovascular interface in the heart. Science (1979) 2023;381:897–906. 10.1126/science.ade4961 37616346

[25] Scherschel K, Bräuninger H, Glufke K, Jungen C, Klöcker N, Meyer C. Location, dissection, and analysis of the murine stellate ganglion. J Vis Exp 2020; doi: 10.3791/62026. 33427236

[26] Olofsson B, Pajusola K, Kaipainen A, von Euler G, Joukov V, Saksela O, Orpana A, Pettersson RF, Alitalo K, Eriksson U. Vascular endothelial growth factor B, a novel growth factor for endothelial cells. Proc Natl Acad Sci U S A 1996;93:2576–2581. 8637916 10.1073/pnas.93.6.2576 PMC39839

[27] Aase K, Lymboussaki A, Kaipainen A, Olofsson B, Alitalo K, Eriksson U. Localization of VEGF-B in the mouse embryo suggests a paracrine role of the growth factor in the developing vasculature. Dev Dyn Off Publ Am Assoc Anat 1999;215:12–25. 10.1002/(SICI)1097-0177(199905)215:1<12::AID-DVDY3>3.0.CO;2-N 10340753

[28] Boon RA, Iekushi K, Lechner S, Seeger T, Fischer A, Heydt S, Kaluza D, Tréguer K, Carmona G, Bonauer A, Horrevoets AJG, Didier N, Girmatsion Z, Biliczki P, Ehrlich JR, Katus HA, Müller OJ, Potente M, Zeiher AM, Hermeking H, Dimmeler S. MicroRNA-34a regulates cardiac ageing and function. Nature 2013;495:107–110. 23426265 10.1038/nature11919

[29] Vidal R, Wagner JUG, Braeuning C, Fischer C, Patrick R, Tombor L, Muhly-Reinholz M, John D, Kliem M, Conrad T, Guimarães-Camboa N, Harvey R, Dimmeler S, Sauer S. Transcriptional heterogeneity of fibroblasts is a hallmark of the aging heart. JCI Insight 2019;4. 10.1172/jci.insight.131092 PMC6948853 31723062

[30] Litviňuková M, Talavera-López C, Maatz H, Reichart D, Worth CL, Lindberg EL, Kanda M, Polanski K, Heinig M, Lee M, Nadelmann ER, Roberts K, Tuck L, Fasouli ES, DeLaughter DM, McDonough B, Wakimoto H, Gorham JM, Samari S, Mahbubani KT, Saeb-Parsy K, Patone G, Boyle JJ, Zhang H, Zhang H, Viveiros A, Oudit GY, Bayraktar OA, Seidman JG, Seidman CE, Noseda M, Hubner N, Teichmann SA. Cells of the adult human heart. Nature 2020;588:466–472. 32971526 10.1038/s41586-020-2797-4 PMC7681775

[31] Schubert M, Klinger B, Klünemann M, Sieber A, Uhlitz F, Sauer S, Garnett MJ, Blüthgen N, Saez-Rodriguez J. Perturbation-response genes reveal signaling footprints in cancer gene expression. Nat Commun 2018;9:20. 29295995 10.1038/s41467-017-02391-6 PMC5750219

[32] Makinen T, Olofsson B, Karpanen T, Hellman U, Soker S, Klagsbrun M, Eriksson U, Alitalo K. Differential binding of vascular endothelial growth factor B splice and proteolytic isoforms to neuropilin-1. J Biol Chem 1999;274:21217–21222. 10409677 10.1074/jbc.274.30.21217

[33] Olofsson B, Pajusola K, von Euler G, Chilov D, Alitalo K, Eriksson U. Genomic organization of the mouse and human genes for vascular endothelial growth factor B (VEGF-B) and characterization of a second splice isoform *. J Biol Chem 1996;271:19310–19317. 8702615 10.1074/jbc.271.32.19310

[34] Rakusan K, Nagai J. Morphometry of arterioles and capillaries in hearts of senescent mice. Cardiovasc Res 1994;28:969–972. 7954608 10.1093/cvr/28.7.969

[35] Sun Y, Jin K, Childs JT, Xie L, Mao XO, Greenberg DA. Vascular endothelial growth factor-B (VEGFB) stimulates neurogenesis: evidence from knockout mice and growth factor administration. Dev Biol 2006;289:329–335. Available from: https://www.sciencedirect.com/science/article/pii/S0012160605007438. 16337622 10.1016/j.ydbio.2005.10.016

[36] Zentilin L, Puligadda U, Lionetti V, Zacchigna S, Collesi C, Pattarini L, Ruozi G, Camporesi S, Sinagra G, Pepe M, Recchia FA, Giacca M. Cardiomyocyte VEGFR-1 activation by VEGF-B induces compensatory hypertrophy and preserves cardiac function after myocardial infarction. FASEB J 2010;24:1467–1478. 20019242 10.1096/fj.09-143180

[37] Oka T, Akazawa H, Naito AT, Komuro I. Angiogenesis and cardiac hypertrophy: maintenance of cardiac function and causative roles in heart failure. Circ Res 2014;114:565–571. 24481846 10.1161/CIRCRESAHA.114.300507

[38] Samuelsson A-M, Bartolomaeus TUP, Anandakumar H, Thowsen I, Nikpey E, Han J, Marko L, Finne K, Tenstad O, Eckstein J, Berndt N, Kühne T, Kedziora S, Sultan I, Skogstrand T, Karlsen TV, Nurmi H, Forslund SK, Bollano E, Alitalo K, Muller DN, Wiig H. VEGF-B hypertrophy predisposes to transition from diastolic to systolic heart failure in hypertensive rats. Cardiovasc Res 2023;119:1553–1567. 36951047 10.1093/cvr/cvad040 PMC10318391

[39] Li J, Tan Y, Passariello CL, Martinez EC, Kritzer MD, Li X, Li X, Li Y, Yu Q, Ohgi K, Thakur H, MacArthur JWJ, Ivey JR, Woo YJ, Emter CA, Dodge-Kafka K, Rosenfeld MG, Kapiloff MS. Signalosome-regulated Serum response factor phosphorylation determining myocyte growth in width versus length as a therapeutic target for heart failure. Circulation 2020;142:2138–2154. 32933333 10.1161/CIRCULATIONAHA.119.044805 PMC7704863

[40] Maillet M, van Berlo JH, Molkentin JD. Molecular basis of physiological heart growth: fundamental concepts and new players. Nat Rev Mol Cell Biol 2013;14:38–48. 23258295 10.1038/nrm3495 PMC4416212

[41] Maroli G, Schänzer A, Günther S, Garcia-Gonzalez C, Rupp S, Schlierbach H, Chen Y, Graumann J, Wietelmann A, Kim J, Braun T. Inhibition of autophagy prevents cardiac dysfunction at early stages of cardiomyopathy in bag3-deficient hearts. J Mol Cell Cardiol 2024;193:53–66. 38838815 10.1016/j.yjmcc.2024.06.001

[42] Lerchenmüller C, Vujic A, Mittag S, Wang A, Rabolli CP, Heß C, Betge F, Rangrez AY, Chaklader M, Guillermier C, Gyngard F, Roh JD, Li H, Steinhauser ML, Frey N, Rothermel B, Dieterich C, Rosenzweig A, Lee RT. Restoration of cardiomyogenesis in aged mouse hearts by voluntary exercise. Circulation 2022;146:412–426. 35862076 10.1161/CIRCULATIONAHA.121.057276 PMC9357140

[43] Yang K-C, Ku Y-C, Lovett M, Nerbonne JM. Combined deep microRNA and mRNA sequencing identifies protective transcriptomal signature of enhanced PI3Kα signaling in cardiac hypertrophy. J Mol Cell Cardiol 2012;53:101–112. Available from: https://www.sciencedirect.com/science/article/pii/S0022282812001599. 22580345 10.1016/j.yjmcc.2012.04.012 PMC3372631

[44] Liu X, Li H, Hastings MH, Xiao C, Damilano F, Platt C, Lerchenmüller C, Zhu H, Wei XP, Yeri A, Most P, Rosenzweig A. miR-222 inhibits pathological cardiac hypertrophy and heart failure. Cardiovasc Res 2024;120:262–272. 38084908 10.1093/cvr/cvad184 PMC10939454

[45] Hou J, Kang YJ. Regression of pathological cardiac hypertrophy: signaling pathways and therapeutic targets. Pharmacol Ther 2012;135:337–354. 22750195 10.1016/j.pharmthera.2012.06.006 PMC3458709

[46] Zeriouh M, Sabashnikov A, Tenbrock A, Neef K, Merkle J, Eghbalzadeh K, Weber C, Liakopoulos OJ, Deppe A-C, Stamm C, Cowan DB, Wahlers T, Choi Y-H. Dysregulation of proangiogeneic factors in pressure-overload left-ventricular hypertrophy results in inadequate capillary growth. Ther Adv Cardiovasc Dis 2019;13:1753944719841795. 31088231 10.1177/1753944719841795 PMC6535753

[47] Burlew BS, Weber KT. Cardiac fibrosis as a cause of diastolic dysfunction. Herz 2002;27:92–98. 12025467 10.1007/s00059-002-2354-y

[48] Reed AL, Tanaka A, Sorescu D, Liu H, Jeong E-M, Sturdy M, Walp ER, Dudley SC, Sutliff RL. Diastolic dysfunction is associated with cardiac fibrosis in the senescence-accelerated mouse. Am J Physiol Circ Physiol 2011;301:H824–H831. 10.1152/ajpheart.00407.2010 PMC3191096 21724869

[49] Raimondi C, Ruhrberg C. Neuropilin signalling in vessels, neurons and tumours. Semin Cell Dev Biol 2013;24:172–178. Available from: https://www.sciencedirect.com/science/article/pii/S1084952113000025. 23319134 10.1016/j.semcdb.2013.01.001

