# Supplementary material for: Beneficial effects of vascular endothelial growth factor B gene transfer in the aged heart
Source: Cardiovasc Res. 2025 Mar 21;121(10):1594–608. doi: 10.1093/cvr/cvaf046 (PMC12391667; doi:10.1093/cvr/cvaf046)
Supplement: cvaf046_Supplementary_Data [file cvaf046_supplementary_data.zip › suppl fig.pptx]

## Slide 1
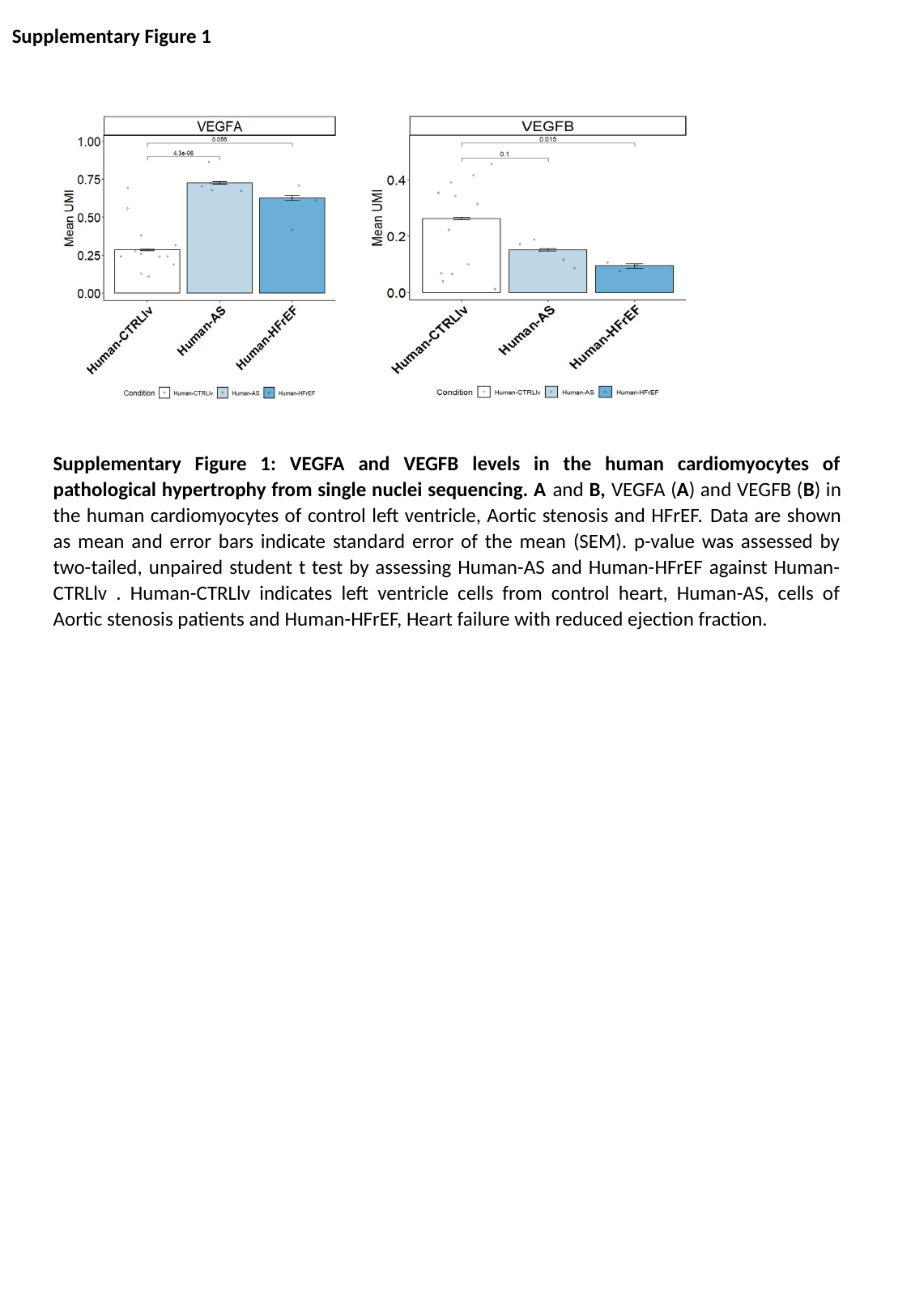

Supplementary Figure 1
Supplementary Figure 1: VEGFA and VEGFB levels in the human cardiomyocytes of pathological hypertrophy from single nuclei sequencing. A and B, VEGFA (A) and VEGFB (B) in the human cardiomyocytes of control left ventricle, Aortic stenosis and HFrEF. Data are shown as mean and error bars indicate standard error of the mean (SEM). p-value was assessed by two-tailed, unpaired student t test by assessing Human-AS and Human-HFrEF against Human-CTRLlv . Human-CTRLlv indicates left ventricle cells from control heart, Human-AS, cells of Aortic stenosis patients and Human-HFrEF, Heart failure with reduced ejection fraction.

## Slide 2
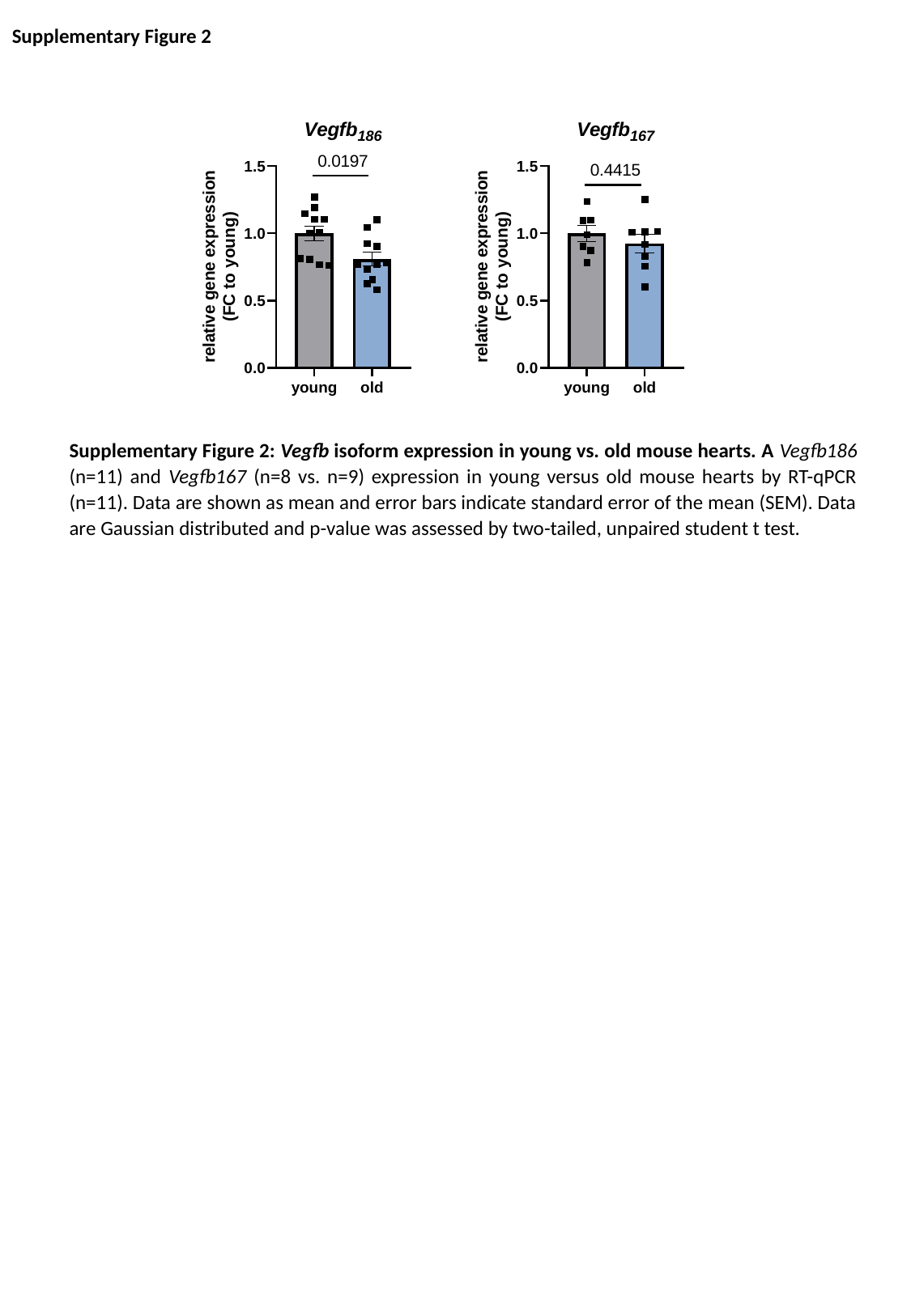

Supplementary Figure 2
Supplementary Figure 2: Vegfb isoform expression in young vs. old mouse hearts. A Vegfb186 (n=11) and Vegfb167 (n=8 vs. n=9) expression in young versus old mouse hearts by RT-qPCR (n=11). Data are shown as mean and error bars indicate standard error of the mean (SEM). Data are Gaussian distributed and p-value was assessed by two-tailed, unpaired student t test.

## Slide 3
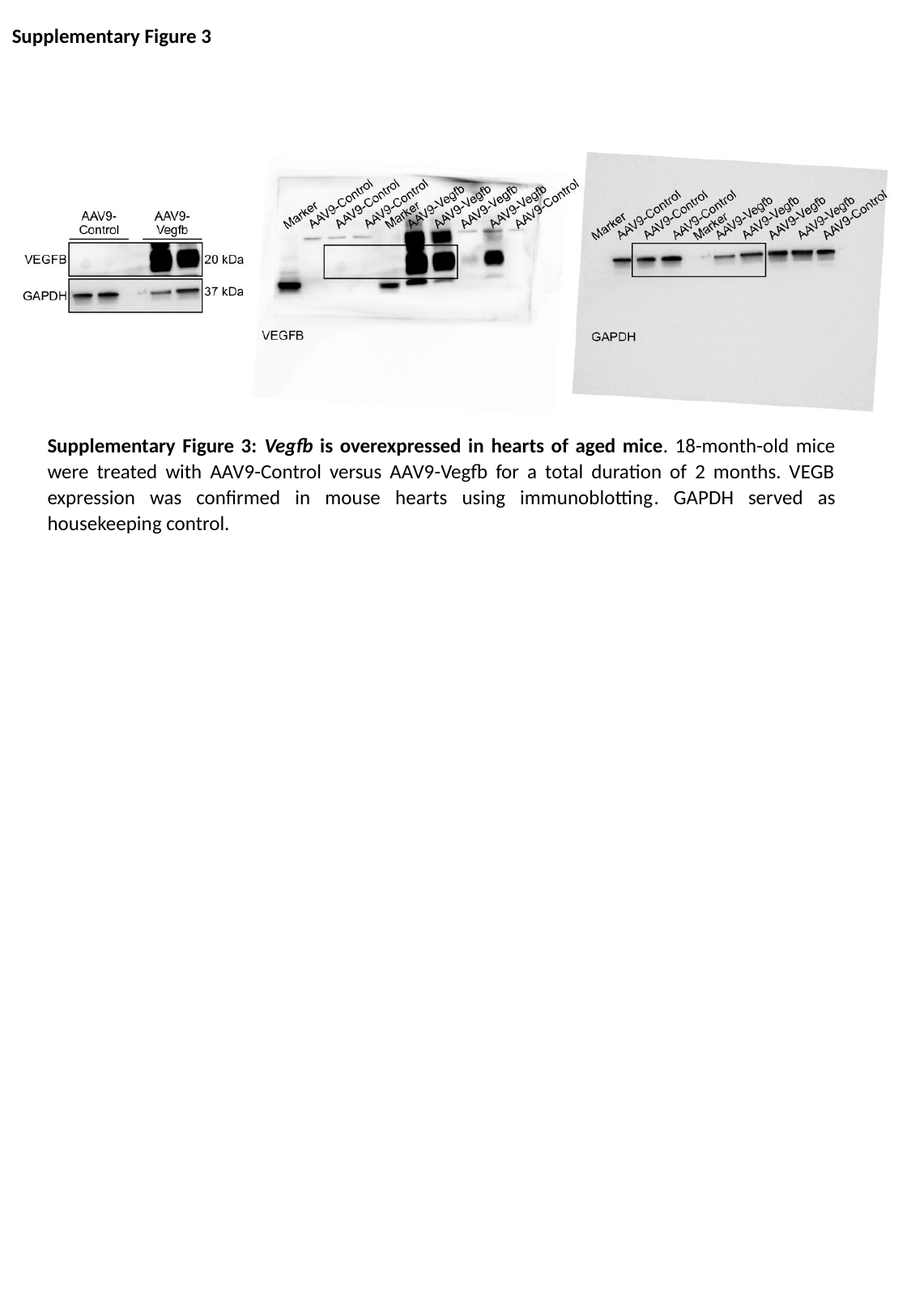

Supplementary Figure 3
Supplementary Figure 3: Vegfb is overexpressed in hearts of aged mice. 18-month-old mice were treated with AAV9-Control versus AAV9-Vegfb for a total duration of 2 months. VEGB expression was confirmed in mouse hearts using immunoblotting. GAPDH served as housekeeping control.

## Slide 4
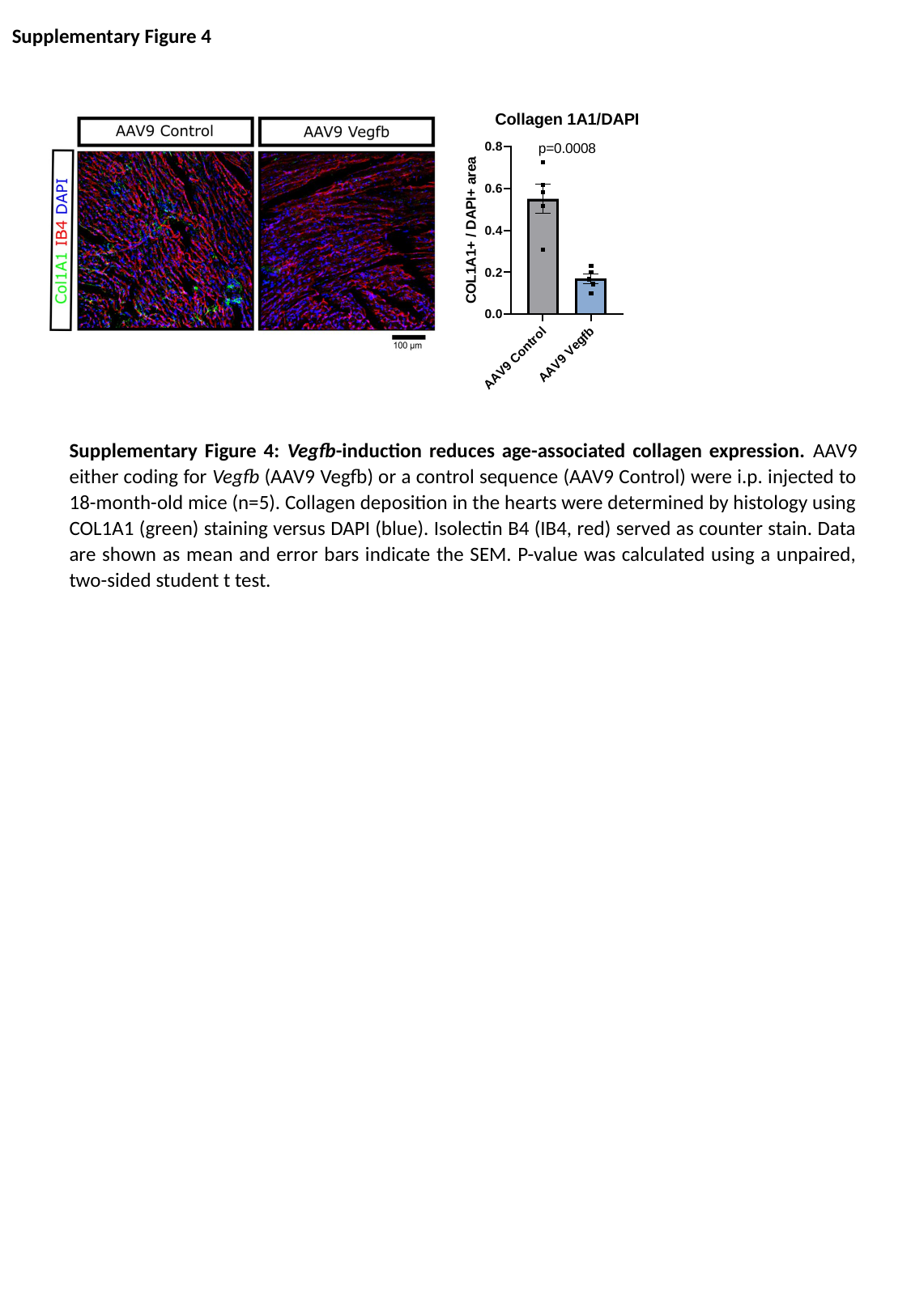

Supplementary Figure 4
Supplementary Figure 4: Vegfb-induction reduces age-associated collagen expression. AAV9 either coding for Vegfb (AAV9 Vegfb) or a control sequence (AAV9 Control) were i.p. injected to 18-month-old mice (n=5). Collagen deposition in the hearts were determined by histology using COL1A1 (green) staining versus DAPI (blue). Isolectin B4 (IB4, red) served as counter stain. Data are shown as mean and error bars indicate the SEM. P-value was calculated using a unpaired, two-sided student t test.

## Slide 5
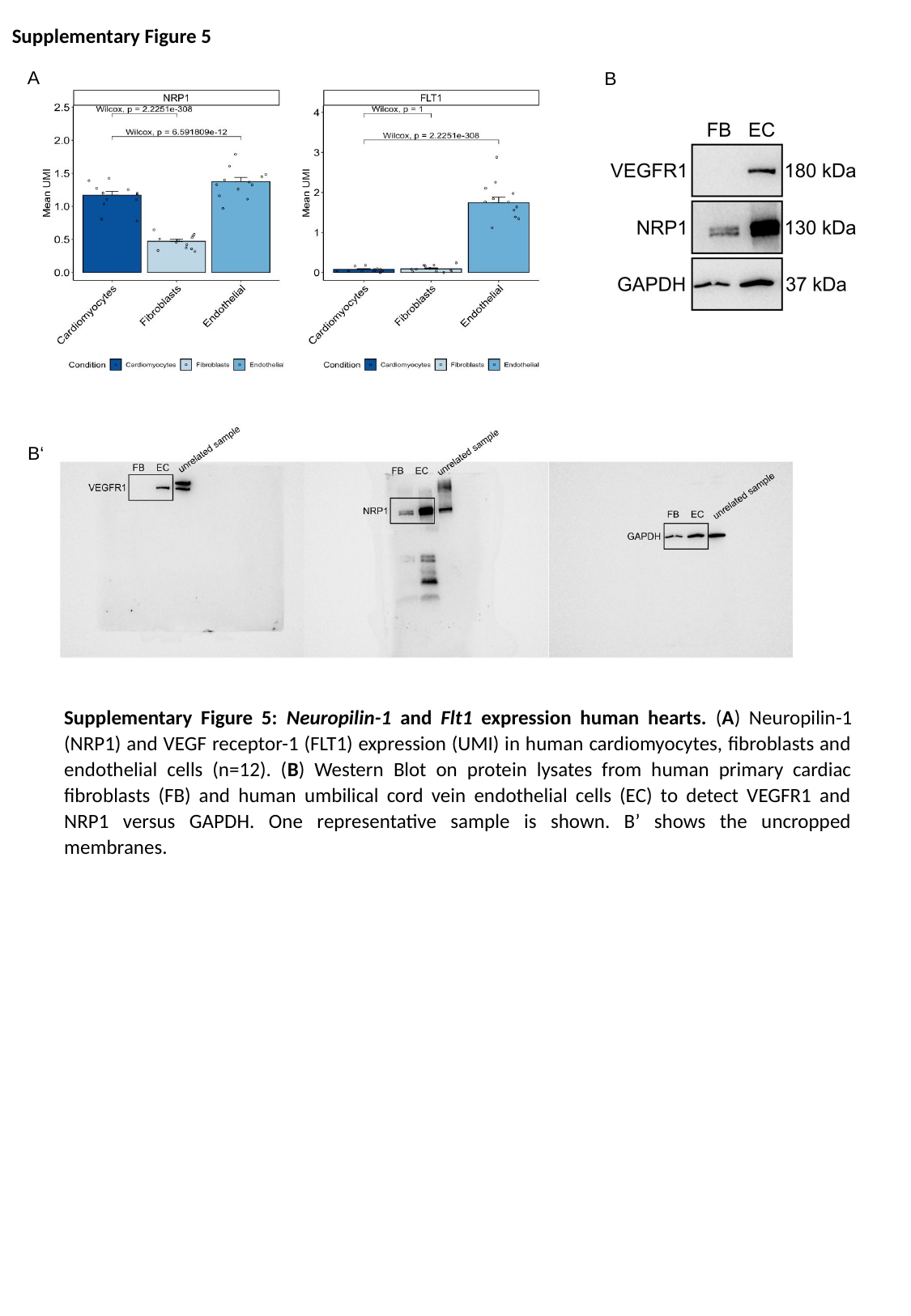

Supplementary Figure 5
A
B
B‘
Supplementary Figure 5: Neuropilin-1 and Flt1 expression human hearts. (A) Neuropilin-1 (NRP1) and VEGF receptor-1 (FLT1) expression (UMI) in human cardiomyocytes, fibroblasts and endothelial cells (n=12). (B) Western Blot on protein lysates from human primary cardiac fibroblasts (FB) and human umbilical cord vein endothelial cells (EC) to detect VEGFR1 and NRP1 versus GAPDH. One representative sample is shown. B’ shows the uncropped membranes.

## Slide 6
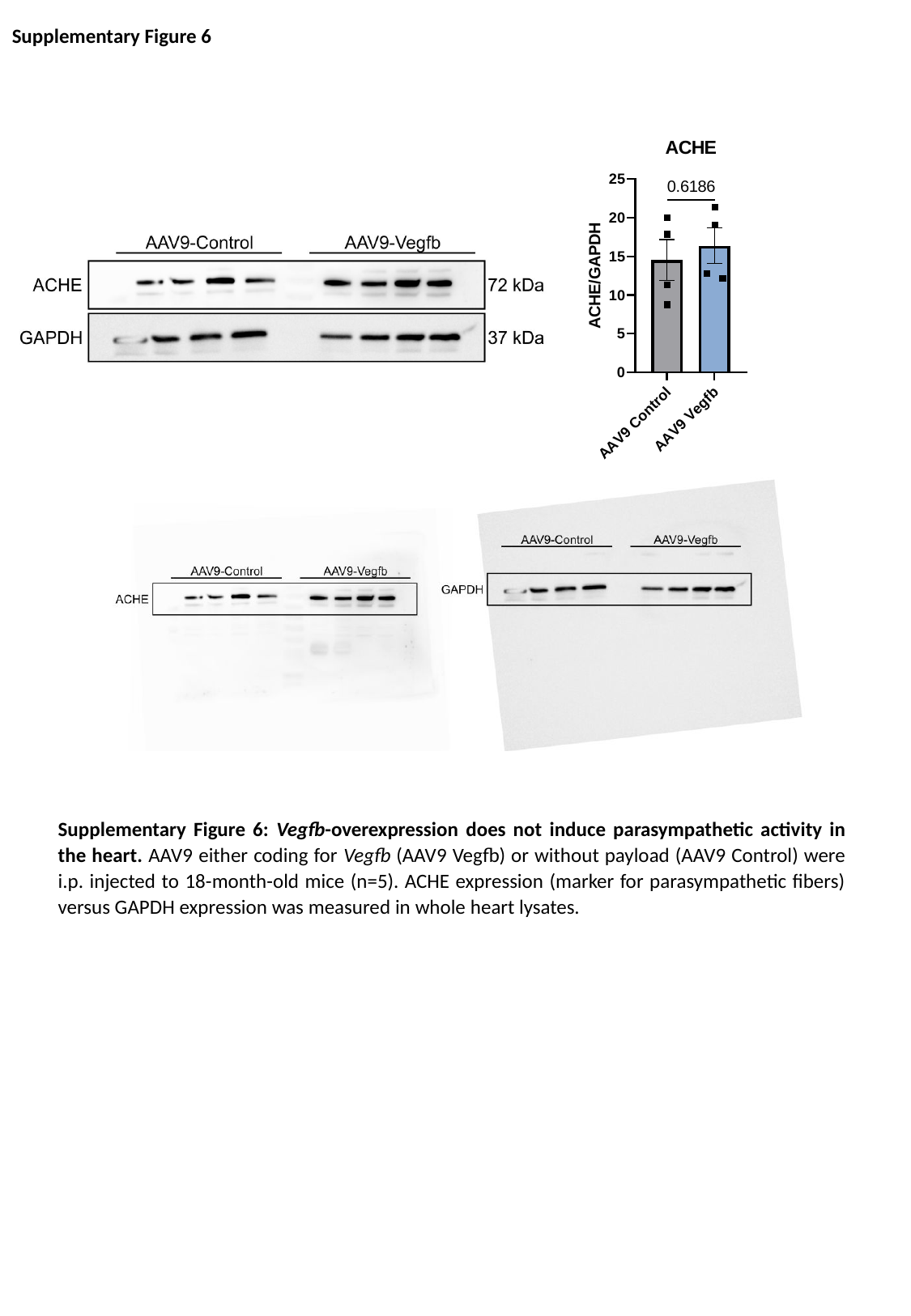

Supplementary Figure 6
Supplementary Figure 6: Vegfb-overexpression does not induce parasympathetic activity in the heart. AAV9 either coding for Vegfb (AAV9 Vegfb) or without payload (AAV9 Control) were i.p. injected to 18-month-old mice (n=5). ACHE expression (marker for parasympathetic fibers) versus GAPDH expression was measured in whole heart lysates.

## Slide 7
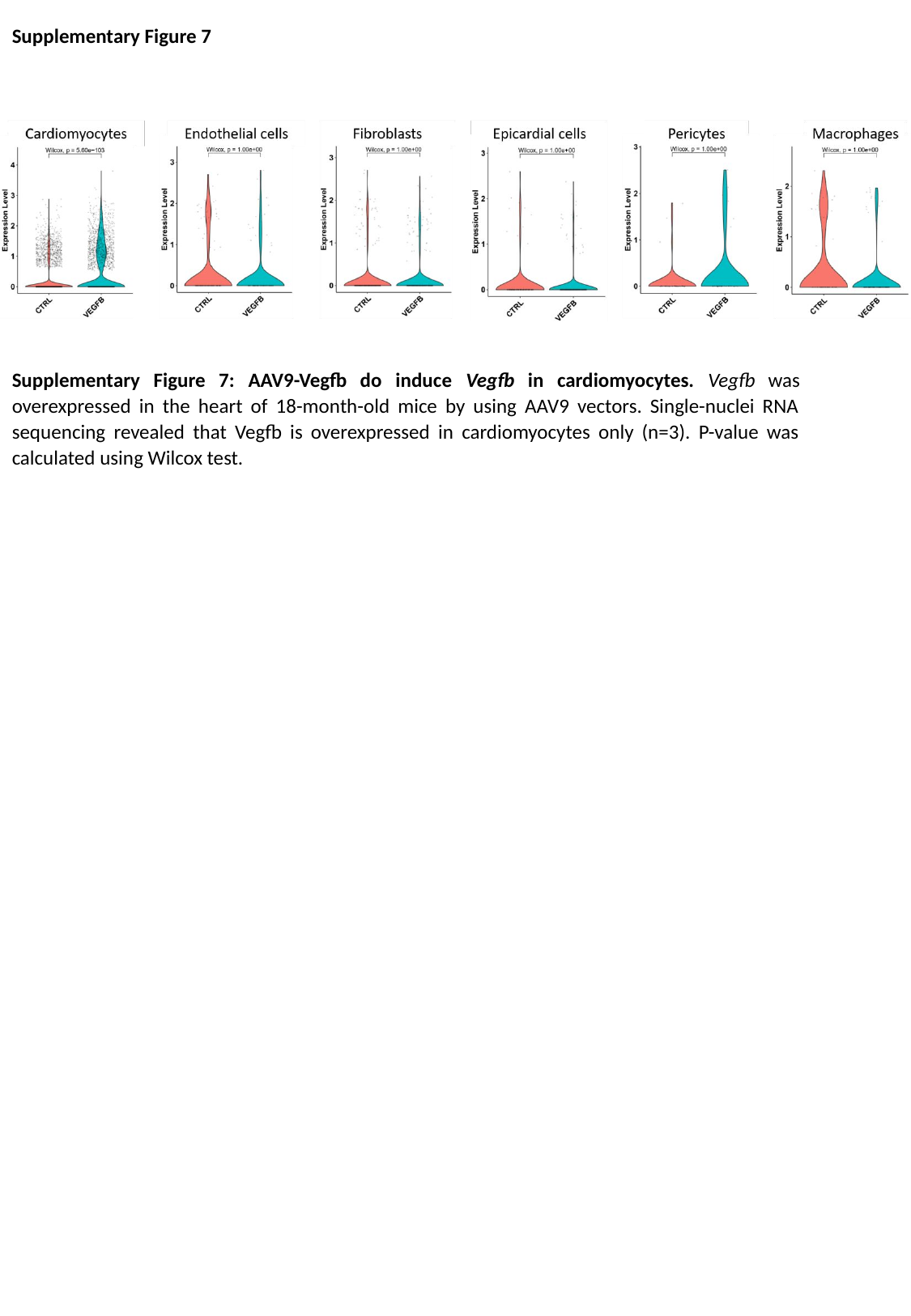

Supplementary Figure 7
Supplementary Figure 7: AAV9-Vegfb do induce Vegfb in cardiomyocytes. Vegfb was overexpressed in the heart of 18-month-old mice by using AAV9 vectors. Single-nuclei RNA sequencing revealed that Vegfb is overexpressed in cardiomyocytes only (n=3). P-value was calculated using Wilcox test.

## Slide 8
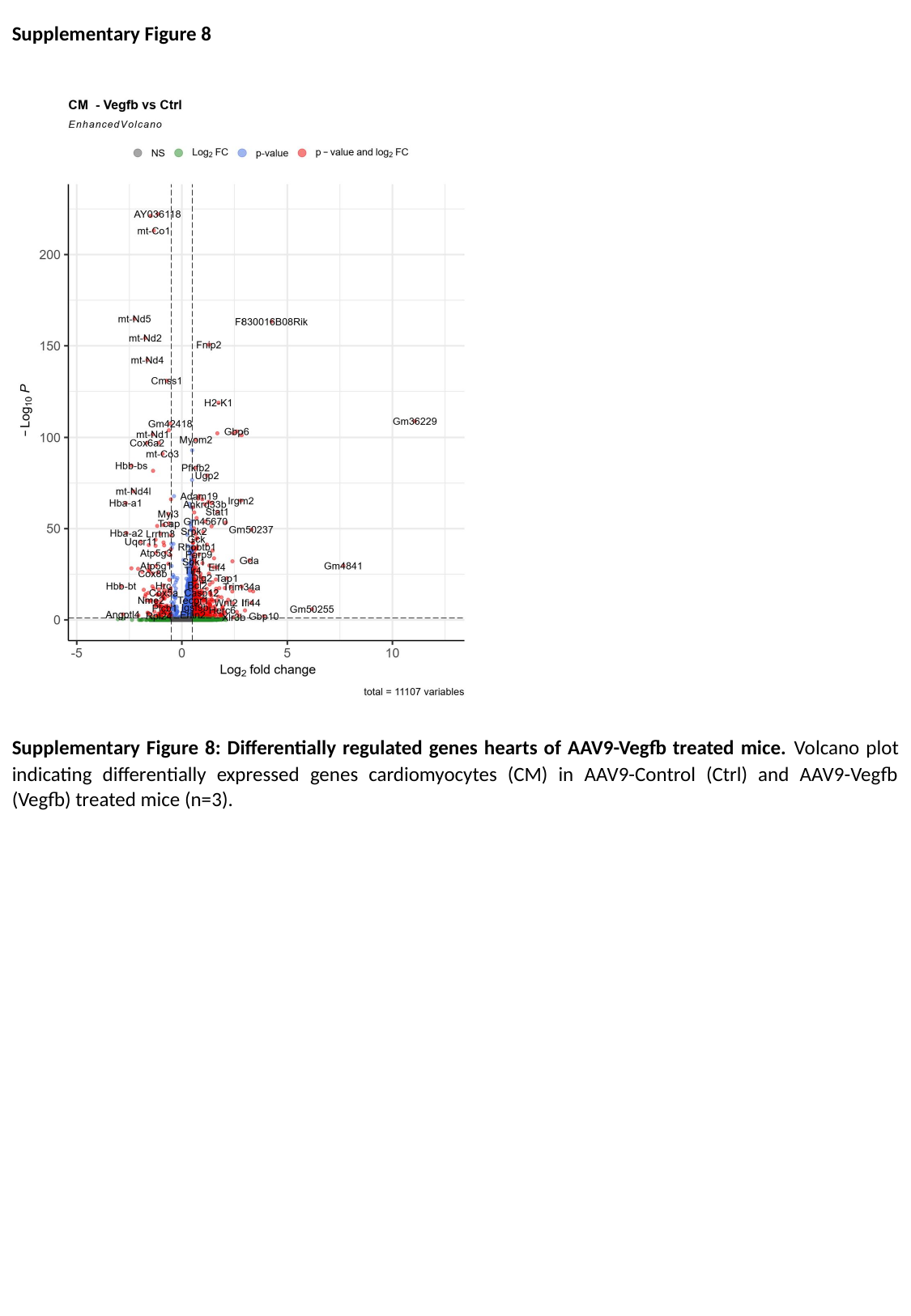

Supplementary Figure 8
Supplementary Figure 8: Differentially regulated genes hearts of AAV9-Vegfb treated mice. Volcano plot indicating differentially expressed genes cardiomyocytes (CM) in AAV9-Control (Ctrl) and AAV9-Vegfb (Vegfb) treated mice (n=3).

## Slide 9
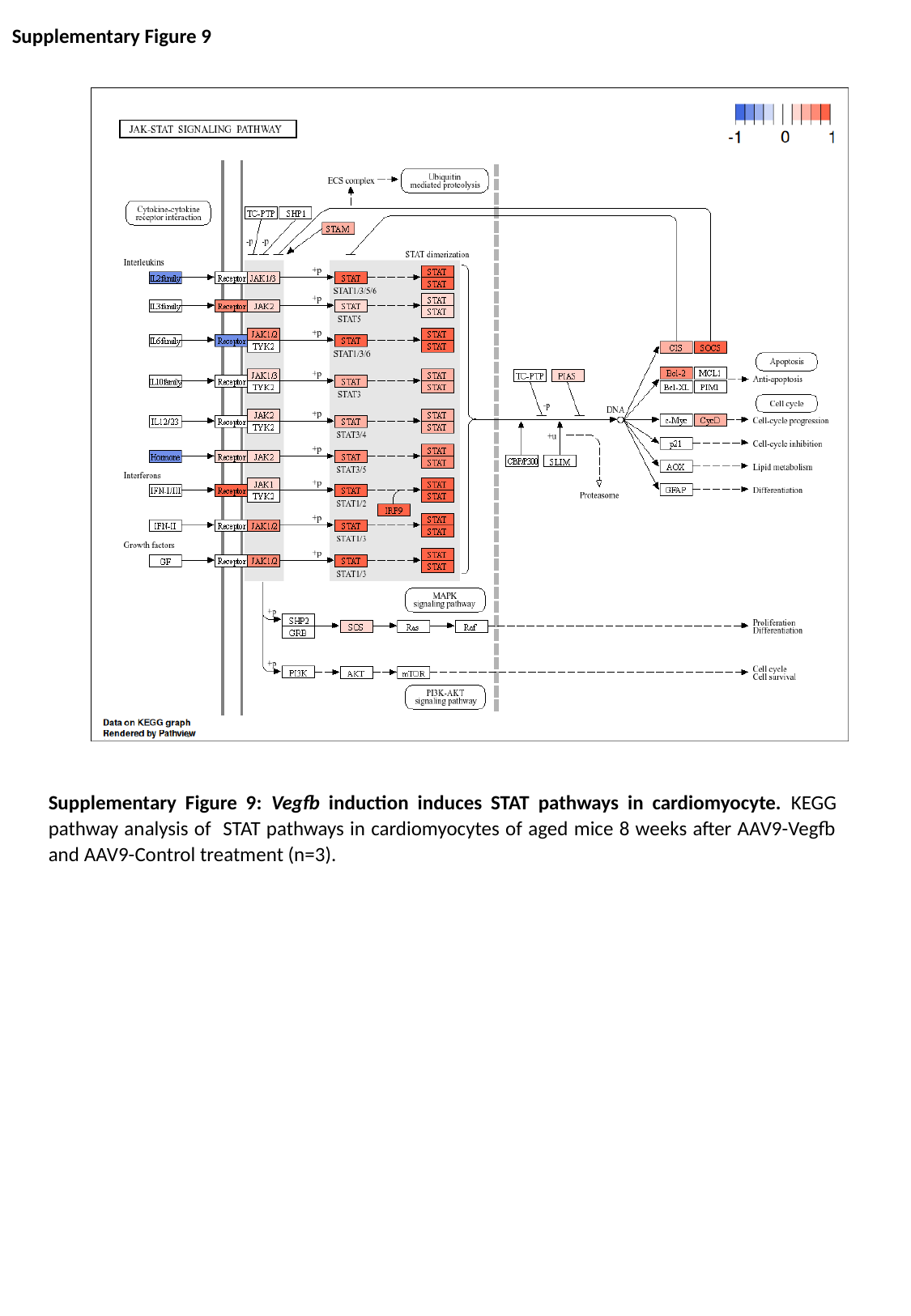

Supplementary Figure 9
Supplementary Figure 9: Vegfb induction induces STAT pathways in cardiomyocyte. KEGG pathway analysis of STAT pathways in cardiomyocytes of aged mice 8 weeks after AAV9-Vegfb and AAV9-Control treatment (n=3).

## Slide 10
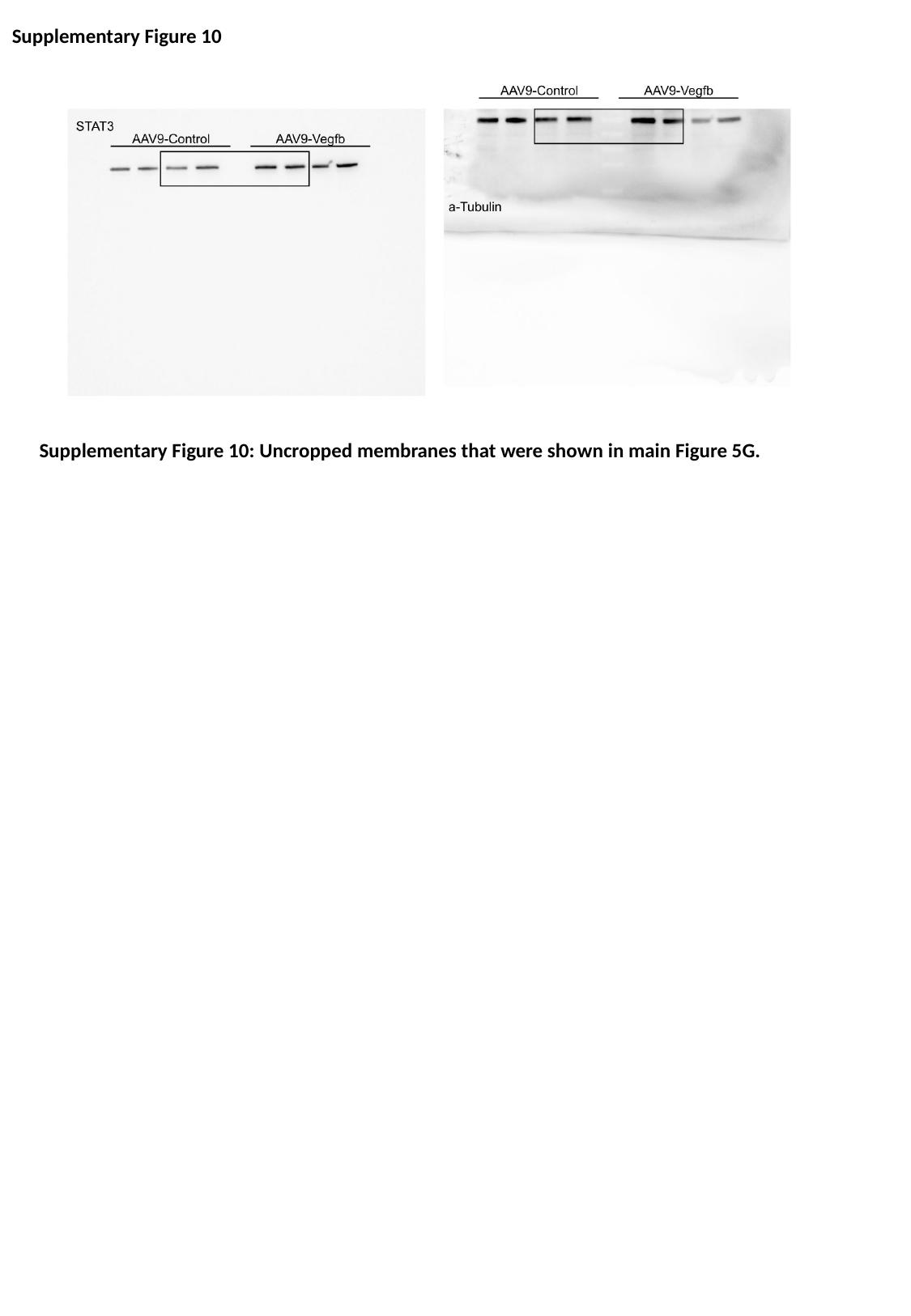

Supplementary Figure 10
Supplementary Figure 10: Uncropped membranes that were shown in main Figure 5G.

## Slide 11
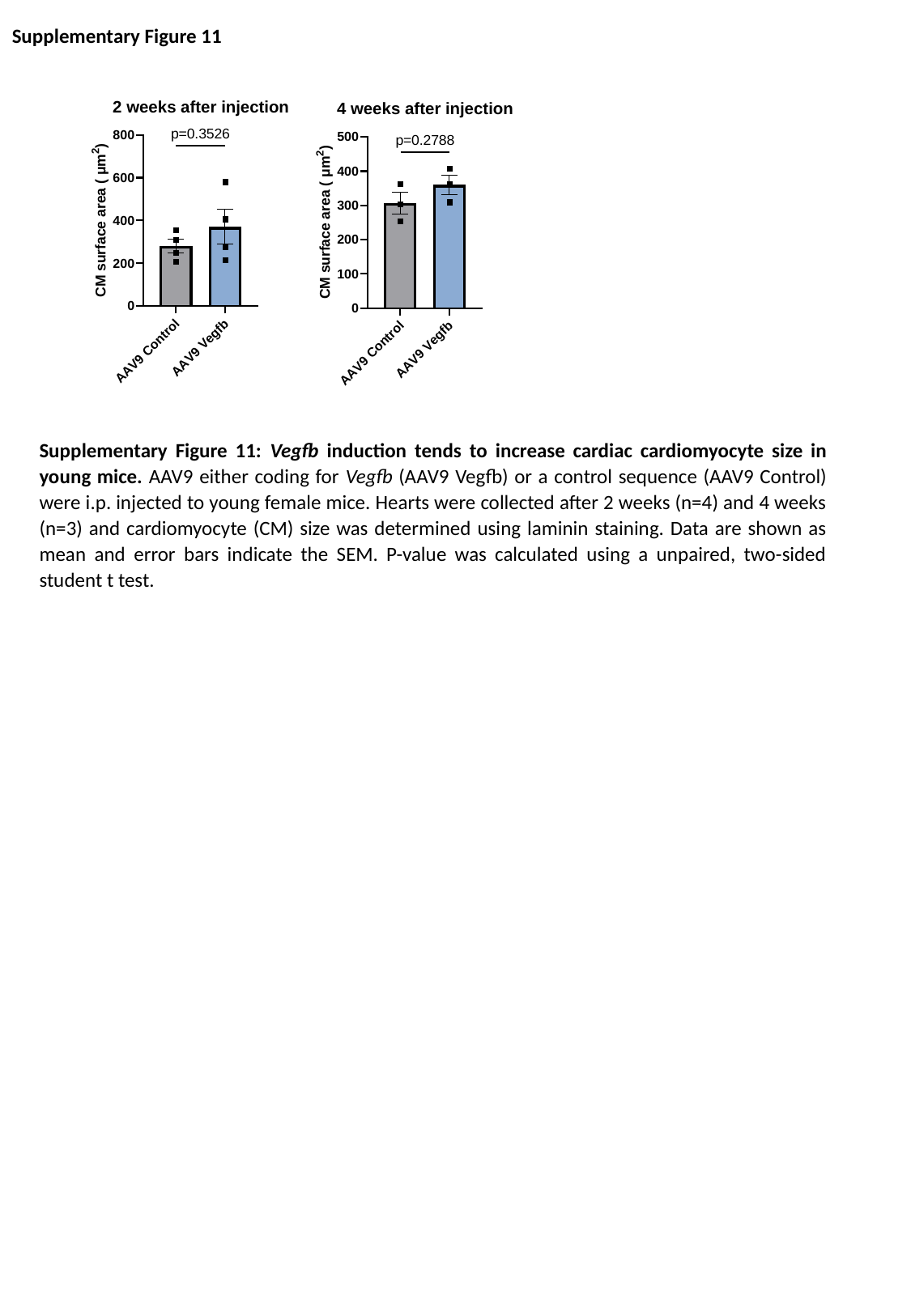

Supplementary Figure 11
Supplementary Figure 11: Vegfb induction tends to increase cardiac cardiomyocyte size in young mice. AAV9 either coding for Vegfb (AAV9 Vegfb) or a control sequence (AAV9 Control) were i.p. injected to young female mice. Hearts were collected after 2 weeks (n=4) and 4 weeks (n=3) and cardiomyocyte (CM) size was determined using laminin staining. Data are shown as mean and error bars indicate the SEM. P-value was calculated using a unpaired, two-sided student t test.

## Slide 12
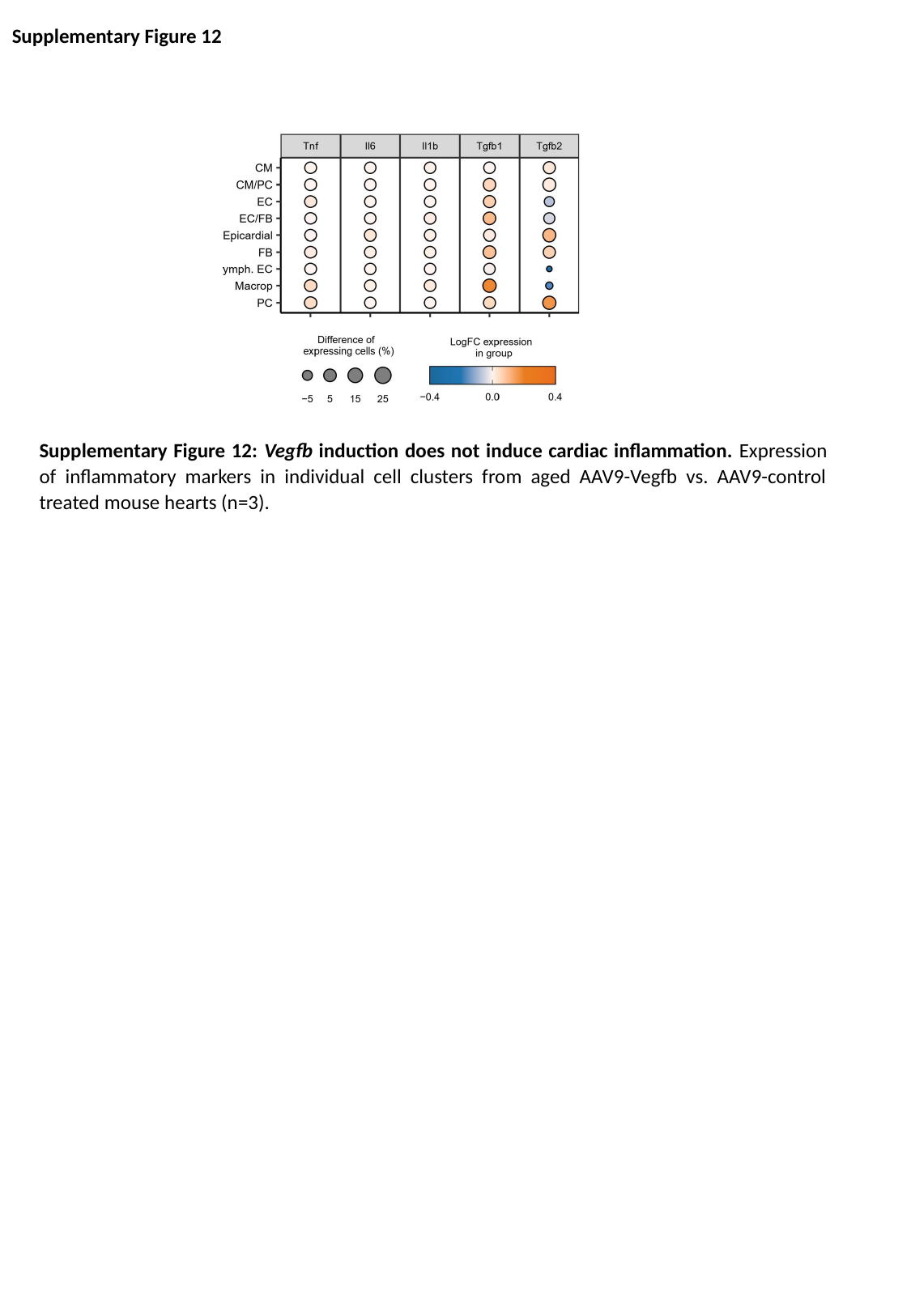

Supplementary Figure 12
Supplementary Figure 12: Vegfb induction does not induce cardiac inflammation. Expression of inflammatory markers in individual cell clusters from aged AAV9-Vegfb vs. AAV9-control treated mouse hearts (n=3).
